# Antibiotic-resistant *Acinetobacter baumannii* can be killed by a combination of bacteriophages and complement

**DOI:** 10.1007/s00430-025-00852-0

**Published:** 2025-09-02

**Authors:** Carmen Chen, Eva Krzyżewska-Dudek, Sheetal Patpatia, Vinaya Dulipati, Sarah Natalia Mapelli, Aycan Meral, Juha Kotimaa, Saija Kiljunen, Seppo Meri

**Affiliations:** 1 Translational Immunology Research Program, Department of Bacteriology and Immunology, Faculty of Medicine, University of Helsinki, Helsinki, Finland; 2 Department of Biomedical Sciences, Humanitas University, Milan, Italy; 3 Department of Immunology of Infectious Diseases, Hirszfeld Institute of Immunology and Experimental Therapy, Polish Academy of Sciences, Wrocław, Poland; 4 Human Microbiome Research Program, Department of Bacteriology and Immunology, Faculty of Medicine, University of Helsinki, Helsinki, Finland; 5 Division of Clinical Microbiology, Department of Laboratory Medicine, Karolinska Institutet, Stockholm, Sweden; 6 Department of Research in Inflammation and Immunology, IRCCS Humanitas Research Hospital, Milan, Italy; 7 Department BA6311 Immunotechnology, VTT Technical Research Centre of Finland, Espoo, Finland; 8 Diagnostic Center (HUSLAB), Helsinki University Hospital, Helsinki, Finland

**Keywords:** Complement system, Bacteriophage, Multidrug-resistant bacteria, Capsular polysaccharides, Transposon mutagenesis, *Acinetobacter baumannii*

## Abstract

Infections caused by multidrug-resistant *Acinetobacter baumannii* are an emerging global health threat. Although phages have shown promising results in treating bacterial infections, the mechanisms of the combined effect of phages and innate immunity on clearing *A. baumannii* remain unclear. Here, we report a synergistic effect of the complement system and phages on clearing multidrug-resistant *A. baumannii*. We show that *A. baumannii* rapidly adapts and becomes resistant to phage or serum complement by modifying the expression of capsule and lipooligosaccharides, which can be regulated through reversible transposon mutagenesis in the K locus. Compared to the encapsulated phenotype, the non-encapsulated, phage-resistant *A. baumannii* showed a higher level of membrane attack complex deposition and were susceptible to killing by complement. In contrast, the encapsulated phenotype escaped the complement system by shedding the membrane attack complex to the environment. Thus, while the complement system targets the non-encapsulated phenotype, the phage infects and eliminates the encapsulated subpopulation. These results suggest means of combatting antibiotic-resistant *A. baumannii* by a simultaneous treatment with phages and complement, a combination which can be supplemented further with antibacterial antibodies.

**Graphical abstract:**

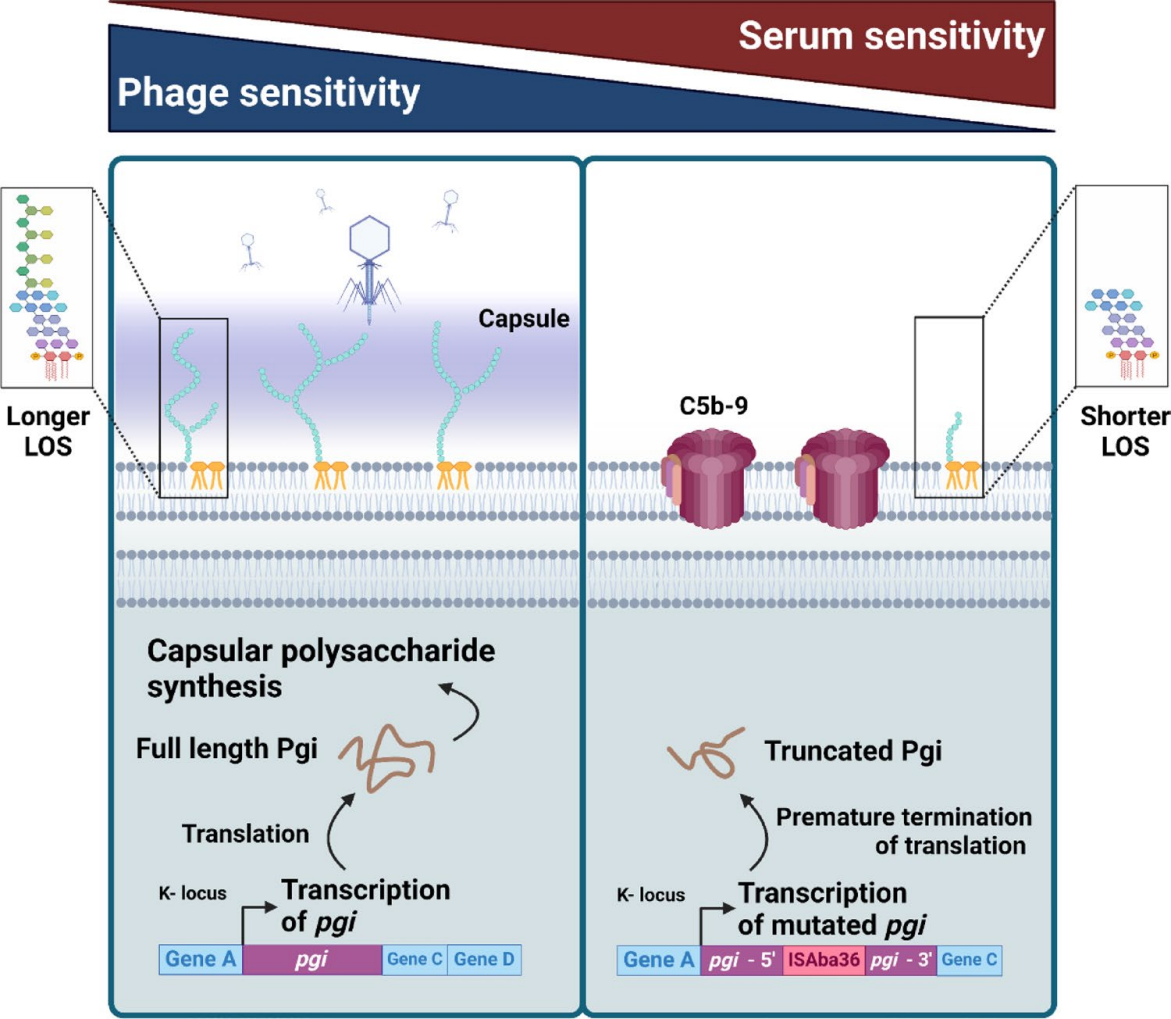

**Supplementary Information:**

The online version contains supplementary material available at 10.1007/s00430-025-00852-0.

## Introduction

*Acinetobacter baumannii* is an opportunistic bacterium, that can cause nosocomial infections and has been ranked under the critical group in the WHO priority list for research and development of new antibiotics [1]. This bacterium can cause pneumonia, meningitis, urinary tract infections, wound infections and sepsis [2, 3]. Immunocompromised patients are particularly vulnerable, and infections caused by *A. baumannii* have become increasingly difficult to treat because of the development of multi-drug resistance [4, 5].

The complement system is part of the human innate immune system and plays a crucial role in defending against bacterial infections by triggering activation of one or more of the three complement pathways: the classical, lectin or alternative pathway. The classical pathway can be activated through the recognition of bacterial surfaces by IgG, IgM or through direct binding of C1q. IgG and IgM can further recruit C1q, which complexes with C1r and C1s to mediate the cleavage of C4 that generates C4a and C4b. When C2 binds to C4b it is cleaved by C1s to C2a and C2b, whereafter the complex of C4b2b constitutes the classical pathway C3 convertase [6]. The lectin pathway can be triggered through the recognition molecules, mannan-binding lectin (MBL) or ficolins that recognise carbohydrates and acetylated moieties on bacterial surfaces, respectively [6, 7]. Through the binding of the recognition molecules with the MBL-associated serine protease-2 (MASP-2), C4 and C2 are cleaved to form C4b2b [7, 8]. Unlike the classical and the lectin pathways, the alternative pathway can be activated through the spontaneous hydrolysis of C3, forming C3(H_2_O) that can bind factor B and generate an initial C3 convertase C3(H_2_O)Bb. This will activate new C3 molecules to C3b that bind factor B. After cleavage of C3b bound B to Bb the actual alternative pathway C3 convertase C3bBb is generated. Eventually, all three pathways converge to activate C3 to the anaphylatoxin C3a and opsonin C3b. The C3-convertases will further activate C5 [6, 9]. Subsequently, C5b recruits C6 to form a soluble C5b6 complex. Upon binding of C7 and C8 the C5b-7 and C5b-8 complexes may already bind and enter into membranes like the outer membranes of gram-negative bacteria. Finally, C9 associates with the C5b-8 complex, unfolds and becomes inserted into the membrane. Further binding of multiple C9 molecules leads to a polymeric C9 ring as part of the complement membrane attack complex (MAC) on the bacterial outer membrane, leading to increased permeability and target cell lysis [6, 10].

Among the many immune evasion mechanisms, some pathogenic bacteria have the ability to resist complement-mediated opsonophagocytosis or killing by hijacking C4BP or factor H [11, 12], which are soluble inhibitors of the C4b2b and C3bBb convertases, respectively [13, 14]. Whether *A. baumannii* is able to escape the complement system by acquiring factor H remains elusive, as contradicting results have been reported [15–17]. However, it is known that *A. baumannii* can survive in normal human serum (NHS) and escape complement through the expression of outer membrane surface proteins CipA and Tuf, production of capsular polysaccharides, modulation of the lipopolysaccharide structures and protein O-glycosylation [17–24].

As antibiotic-resistant *A. baumannii* have emerged and become increasingly prevalent, alternative therapies are urgently needed. Bacteriophages (phages), which are bacterial viruses, have shown their potential as an alternative therapeutic means to antibiotics [25–27]. Phages can kill their host bacterial cells by undergoing a lytic cycle, where the phages infect and propagate within the host by utilising its metabolic machinery. Eventually, the bacterial host cell is lysed upon the release of phage particles into the environment [28, 29]. However, as bacteria and phages are coevolving, phages can rapidly drive the selection of bacteria that are resistant to phages [29, 30]. This complicates the therapeutic application of phages to cure bacterial infections. Moreover, the fact that phagocytes and serum components, such as antibodies and C1q, can clear and inactivate phages, raises concerns about the efficacy of phages to infect and kill bacteria in the human body [31–34]. Although phages can be phagocytosed and neutralised in serum, they may induce an alternative bacterial killing pathway within macrophages [35], or together with complement clear the bacteria in a synergistic manner [36].

In our previous work, we isolated *A. baumannii*-specific phages belonging to the family *Autographviridae* [37]. In this work we demonstrate that *A. baumannii* can rapidly adapt and become resistant to phages or complement-mediated killing by undergoing morphological changes. Notably, even though *A. baumannii* may exist as heterogenous bacterial populations with distinct characteristics, our findings show that phages and the complement system have a synergistic effect on the killing of *A. baumannii*, a feature that could be exploited therapeutically.

## Materials and methods

### Ethics statement

The work was carried out under Helsinki University Hospital research permits for projects TYH2022315 and TYH202322. Written informed consents were obtained from each healthy blood donor. The work does not involve handling or storing patient samples such as tissues, blood, or cells, nor patient personal data. The bacterial strains used in the work have been obtained as pure cultures as anonymised and named based on their sequential storage number in the strain collection of the University of Helsinki research group.

### Serum collection

Pooled normal human serum (NHS) was collected from 8 to 16 healthy donors. All donors provided a written consent. After allowing the drawn blood to clot, it was centrifuged at 1200 rcf for 10 min. Equal volumes of sera from females and males were pooled, and complement activity was preserved by storage at − 80 °C. Pooled heat-inactivated serum (HIS) was prepared by incubating NHS at 56 °C for at least 30 min.

### Bacteria and bacteriophage strains

The clinical *A. baumannii* 5542, 5707 and 5910 strains were isolated at the Helsinki University Central Hospital Diagnostic Center (HUSLAB). *Acinetobacter* viruses fBenAci001, fBenAci002 and fBenAci003, which infect *A. baumannii* 5542, 5707 and 5910 respectively, were isolated from Beninese wastewater [37]. Production of phage lysates has been previously described [38, 39]. A phage-resistant *A. baumannii* 5910 strain (5910-R_φ_) was isolated from a single colony grown on a plaque through the double-layer agar plate method, as described earlier [38]. 5910-R_φ_ was incubated in Lysogeny broth (LB) containing 25% NHS overnight at 37 °C. Thereafter, the culture was passaged once more to LB with 25% NHS before plating the bacteria on an LB plate. A single colony of the survivors was isolated (5910-S_φ_). The serum- and phage-sensitivities of 5910-R_φ_ and 5910-S_φ_ were tested by determining the bacterial growth curves and using the double layer-agar method. All bacterial strains were grown in LB medium at 37 °C, with shaking unless stated otherwise. Bacteria and bacteriophages were stored in 30% glycerol and 8% DMSO, respectively, at − 80 °C. Detailed information regarding the phenotype of *A. baumannii* 5910 strain and the BioSample accession numbers are summarised in Table S1.

### Viability assay for *A. baumannii* and detection of heterogeneous bacterial populations

Measurement of bacterial growth curves was performed as described previously with some modifications [39]. Briefly, phage lysates were diluted in Tris^++^ buffer [10 mM Tris–HCl, 150 mM NaCl, 2 mM CaCl_2_, 0.5 mM MgCl_2_, pH = 7.3]. Phage fBenAci001 (1 × 10^6^ plaque-forming units (PFU)), fBenAci002 (1 × 10^4^ PFU) or fBenAci003 (1 × 10^6^ PFU) was added into wells of a honeycomb plate with or without HIS or NHS. Overnight bacterial cultures were refreshed in LB medium and incubated until the mid-exponential phase was reached (OD_600_ = 0.5 – 0.7). *A. baumannii* 5542 strain (1 × 10^6^ CFU), 5707 strain (1 × 10^6^ CFU) or 5910 strain (1 × 10^4^ CFU) were seeded into each well, and the final serum concentration was 25% after the addition of bacteria. Bacteria were grown in the honeycomb plate at 37 °C with shaking in a Bioscreen C analyzer (Growth Curves AB Ltd), and the bacterial growth was followed for 23 h by monitoring the absorbance at 600 nm every 20 min. Similarly, for the detection of a heterogeneous bacterial population, the growth of three single colonies of the parental 5910 strain was monitored by measuring the absorbance at 600 nm. Ten million bacteria from a refreshed culture were seeded into a well that either contained LB and Tris^++^, phage fBenAci003 (1 × 10^6^ PFU), HIS or NHS, with a final serum concentration of 25%. Measurement of the growth curves of preconditioned parental 5910 strain were performed in similar way after refreshing the overnight bacterial cultures in LB. To precondition the parental 5910 strain, a single colony was inoculated in LB supplemented with 25% HIS, 25% NHS or 10 µl of phage lysates (10^7^–10^8^ PFU/ml) and incubated overnight at 37 °C.

### Rescue assay of serum-sensitive bacteria

NHS or HIS were incubated with 100 µg/ml ravulizumab (Alexion), a monoclonal complement C5 blocking antibody, at room temperature for 35 min. Ten million cells of refreshed 5910-R_φ_ bacterial cultures in LB and Tris^++^ were seeded into each well. The final serum concentration was 25% after the addition of bacteria. Bacteria were grown at 37 °C with continuous shaking and the growth was monitored every 20 min for 24 h with the BioScreen C analyzer at 600 nm.

### Capsule staining

Overnight bacterial cultures were pelleted and mixed with 10 µl of India ink (Lefranc & Bourgeois) on a glass slide. The bacteria-ink solution was spread on the glass slide and air-dried before flooding the slides with 1% crystal violet for 2 min. Excess crystal violet was removed by tilting and blotting the edges of the slides. A coverslip was mounted on top of the stained area while the slides were still wet. Stained bacteria were visualised under Olympus-BX51 fluorescent microscope with bright field at 100 × magnification. All images were acquired with the Olympus DP-70 camera attached to the microscope. The brightness and contrast of all images were adjusted by using Microsoft PowerPoint.

### Isolation and staining of capsular polysaccharides

Bacterial capsular polysaccharides were isolated as described earlier [40] with minor modifications. Briefly, bacteria were incubated overnight at 37 °C, 220 rpm in LB medium that was either supplemented with 25% HIS, 25% NHS, or 10 µl of phage lysate (10^7^–10^8^ PFU/ml). Overnight cultures were reinoculated in 20 ml of LB without any supplements and incubated at 37 °C, 230 rpm overnight. The pellet of overnight bacterial cultures was collected after centrifugation at 4690 rcf for 30 min, followed by a wash in saline and centrifugation. Capsular polysaccharides were extracted by resuspending bacterial pellets in 50 mM citrate buffer with 0.1% (v/w) n-tetradecyl-n,n-dimethyl-3-ammonio-1-propanesulfonate (zwitterreagent, Merck), pH 4.5, followed by incubation at 42 °C, 900 rpm for 40 min. The volume of added citrate buffer was adjusted according to the wet mass of the bacterial pellet to a concentration of 0.1 g/ml. Samples were centrifuged at 16,000 rcf for 5 min, and the supernatants were collected. Samples were separated on 4–20% Bis–Tris gels (Invitrogen) at 165 V, 130 mA for 35 min with MES running buffer (Invitrogen). The gel was washed with Milli-Q water for 30 min and stained with 0.1% (w/v) Alcian blue in 40% ethanol/ 60% 20 mM sodium acetate with a pH of 4.75 for 1 h at ambient temperature, followed by overnight incubation in 40% ethanol/60% of 20 mM sodium acetate with a pH of 4.75 at ambient temperature to de-stain the gel. The gel was imaged under white light using a GelDoc XR imaging system (Bio-Rad).

### Lipooligosaccharide isolation and silver staining

Bacteria were cultivated at 37 °C, 220 rpm in LB or LB supplemented with 25% HIS, 25% NHS, or 10 µl of phage lysate (10^7^–10^8^ PFU/ml). Overnight cultures were reinoculated in 50 ml of LB without any supplements and further incubated at 37 °C, 220 rpm overnight. The bacteria were harvested by centrifugation, and lipooligosaccharide (LOS) was extracted according to Galanos et al. [41]. The extraction solution that was used contained aqueous phenol (90%), chloroform, and petroleum ether (PCP, b.p. 40–60 °C) mixed in a volume ratio of 2:5:8. Solid phenol was added in small portions to the mixture until a clear solution was obtained. Dry bacterial mass (100 mg) was placed in a 5 ml Eppendorf tube and 1 ml of PCP solution was added. The mixture was stirred vigorously to a fine suspension at ambient temperature. Next, the cell suspension was centrifuged at 1500 rcf for 15 min. Supernatant was collected and a second PCP extraction was performed. The pooled supernatants from two extractions were evaporated under a stream of N_2_ at 40 °C. Small portions of distilled water were added until LOS was precipitated. Next, the mixture was centrifuged at 20,000 rcf for 20 min, and the supernatant decanted. The precipitate was washed 3 times with 200 µl of 80% phenol, followed by 3 washes with 200 µl of acetone. After the last washing step, the sample was lyophilised. Dry samples were dissolved in distilled water by warming at 80 °C for 2 min and brief sonication. Sample of 12.5 µg was analysed with SDS-PAGE by separation on 15% polyacrylamide gel. The separated LOS was visualised using silver staining according to Tsai and Frasch, and Fomsgaard et al. [42, 43], with minor modifications [44]. The gel was imaged under white light using a GelDoc XR imaging system (Bio-Rad).

### Deposition of C5b-9 on bacterial surface and permeability staining

Bacteria were inoculated in LB medium supplemented with 10 µl of phage lysate (10^7^–10^8^ PFU/ml), 25% HIS or 25% NHS and incubated at 37 °C overnight with shaking. Overnight bacterial cultures were refreshed in LB medium and further cultivated until the mid-exponential phase was reached. For C5b-9 deposition and permeability staining, bacteria were harvested and washed with Tris^++^, whereafter 3 × 10^7^ bacterial cells were seeded into a 96-well plate. Bacteria were incubated with 25% HIS or NHS at 37 °C for 30 min. Half of the bacteria-serum suspension was prepared for C5b-9 staining, and the other part was collected for permeability staining. For C5b-9 staining, the bacterial pellet was mixed with 5 µg/ml mouse-anti-human-C5b-9 neoepitope mAb (clone aE11, Hycult) and incubated for 45 min at ambient temperature. The samples were further incubated with a secondary Alexa-488-labeled goat-anti-mouse-IgG antibody (Invitrogen) at ambient temperature for 45 min. After washing, the bacteria were fixed with 1% paraformaldehyde (PFA) at ambient temperature for 15 min. To assess cell permeability, 100 µl of sample was stained with 7-AAD (Invitrogen) according to the product description provided by the manufacturer. The bacteria were stained in a similar way for the detection of C5b-9 after treatment with 25% NHS for 3, 5, 10 and 30 min, and the supernatant were collected for soluble C5b-9 detection. Samples were analysed using a CytoFLEX flow cytometer (Beckman Counter). To compare C5b-9 deposition between the different phenotypes from independent experiments, the geometric mean fluorescence intensity (gMFI) was normalised according to the level of C5b-9 deposited on 5910-R_φ_ clone.

To inhibit the classical and the lectin pathway, the bacteria were resuspended in Mg-EGTA buffer [10 mM Tris–HCl, 13.33 mM EGTA, 150 mM NaCl, 7 mM MgCl_2_, pH = 7.3] and were seeded in a 96-well plate. The bacteria were incubated with 25% NHS in the Mg-EGTA buffer at 37 °C for 3, 5, 10 and 30 min. Supernatant were collected for soluble C5b-9 detection, and the pellet were stained against C5b-9 and bound antibodies were detected as described above.

### Detection of soluble C5b-9

Bacteria were cultivated in LB supplemented with 10 µl of phage lysate (10^7^–10^8^ PFU/ml), 25% HIS or 25% NHS at 37 °C overnight with shaking. Overnight bacteria cultures were refresh in LB without supplements until the mid-exponential phase was reached. After washing the bacteria with Tris^++^, 3 × 10^7^ bacterial cells were seeded into each well of a 96-well plate. Bacteria were incubated in 25% NHS for 3, 5, 10 and 30 min at 37 °C and the supernatant was collected after the reaction was terminated with EDTA (final concentration 10 mM). Soluble C5b-9 in the supernatant was measured with MicroVue SC5b-9 Plus EIA (Quidel) by following the instructions provided by the manufacturer.

### Isolation of bacterial DNA, whole genome sequencing and genomic analysis

Bacterial DNA was isolated with the JetFlex Genomic DNA Purification Kit (Thermo Fisher Scientific) following the protocol provided by the manufacturer. The quality of the isolated DNA was tested with a Qubit 4 machine (Invitrogen) and 1% agarose electrophoresis. Whole-genome sequencing of 5910, 5910-R_φ_ and 5910-S_φ_ were carried out by Novogene (UK) Co., Ltd by using Illumina Sequencing PE150 and PacBio Sequel II to obtain short and long reads, respectively. The A5-miseq pipeline was used to assemble the obtained Illumina sequences [45]. Hybrid assembly was conducted using Unicycler v0.4.8 [46]. The 5910-R_φ_ and 5910-S_φ_ clones were compared to the parental 5910 strain, and the reads were mapped to a reference genome with BWA-mem and variants were called with Freebayes. The comparisons were conducted on PATRIC by using the variant calling tool [47]. Rapid annotation subsystems technology (RAST) was used for the annotation of the bacterial genome [48–50]. The O- and K-serotypes of *A. baumannii* were determined by Kaptive [51, 52]. Insertion sequence was identified by BLAST [53] and ISfinder [54]. Mutation within the K locus of the *A. baumannii* strains was identified after predicting the K-serotype of the bacterial strains using the Kaptive service. To verify the insertion sequence within glucose-6-phosphate isomerase (*pgi*), the local insertion region was amplified with Phusion High-Fidelity DNA Polymerase (Thermo Fisher Scientific) and the primer pairs gpi_IS_For (5′-TAGTTCAGCTGAATCCAAAG-3′) and gpi_IS_Rev (5′-TACTCCATCAAGGAACTCAG-3′). PCR products were isolated from 2% agarose gels with the GenElute Gel Extraction Kit (Merck). Sanger sequencing of the isolated PCR products was carried out by the Institute for Molecular Medicine Finland.

### RNA isolation and sequencing

The parental *A. baumannii* 5910 strain was cultivated in LB or LB supplemented with phage lysate, 25% HIS or NHS, while 5910-R_φ_ and 5910-S_φ_ were inoculated in LB only. Bacteria were incubated at 37 °C, 220 rpm overnight. On the next day, the overnight cultures were refreshed in LB medium without any supplements and harvested after the bacteria reached the exponential phase. RNAprotect Bacteria Reagent (QIAGEN) was used to stabilise the bacterial RNA, before the bacterial pellets were stored at − 80 °C. The bacteria were lysed with TRIzol Reagent (Invitrogen), and the RNA was isolated with the RNeasy kit according to the protocol provided by the manufacturer (QIAGEN). The quality control and the Illumina Sequencing PE150 of the samples were carried out by Novogene (UK) Co., Ltd. To analyse the expression level of genes in 5910 clones, the sequencing reads were aligned against *A. baumannii* H32 (NCBI RefSeq assembly GCF_030009215.1) using the Chipster platform with the BowtieTie2 pipeline [55, 56]. Differential gene expression was analysed with DESeq2 package (Bioconductor) in Rstudio [57]. Genes were prefiltered, removing those with a raw coverage less than 10 reads. Subsequently, the genes were normalised with variance stabilising transformation (VST). Wald test was computed with the same library. Genes with a log twofold change larger than 1 or less than − 1, and with a padj-value less than 0.05 were considered to be expressed with a significant difference.

### Prediction of depolymerase activity

The DNA sequences of the phage tail spike proteins were uploaded to PhageDPO Phage Depolymerase Finder (Galaxy server of University of Minho, Version 0.1.0) to predict the depolymerase activity [58, 59].

### Quantification and statistical analysis

Deposition of C5b-9 on the bacterial surface and changes in permeability were analysed with FlowJo. Computation of area under the curves and statistical analyses were performed with GraphPad Prism 9. Mann–Whitney and One-Way ANOVA with Kruskal–Wallis tests were used for the analyses.

## Results

### Synergistic effect of complement and bacteriophages on killing of *A. baumannii*

**Fig. 1:**
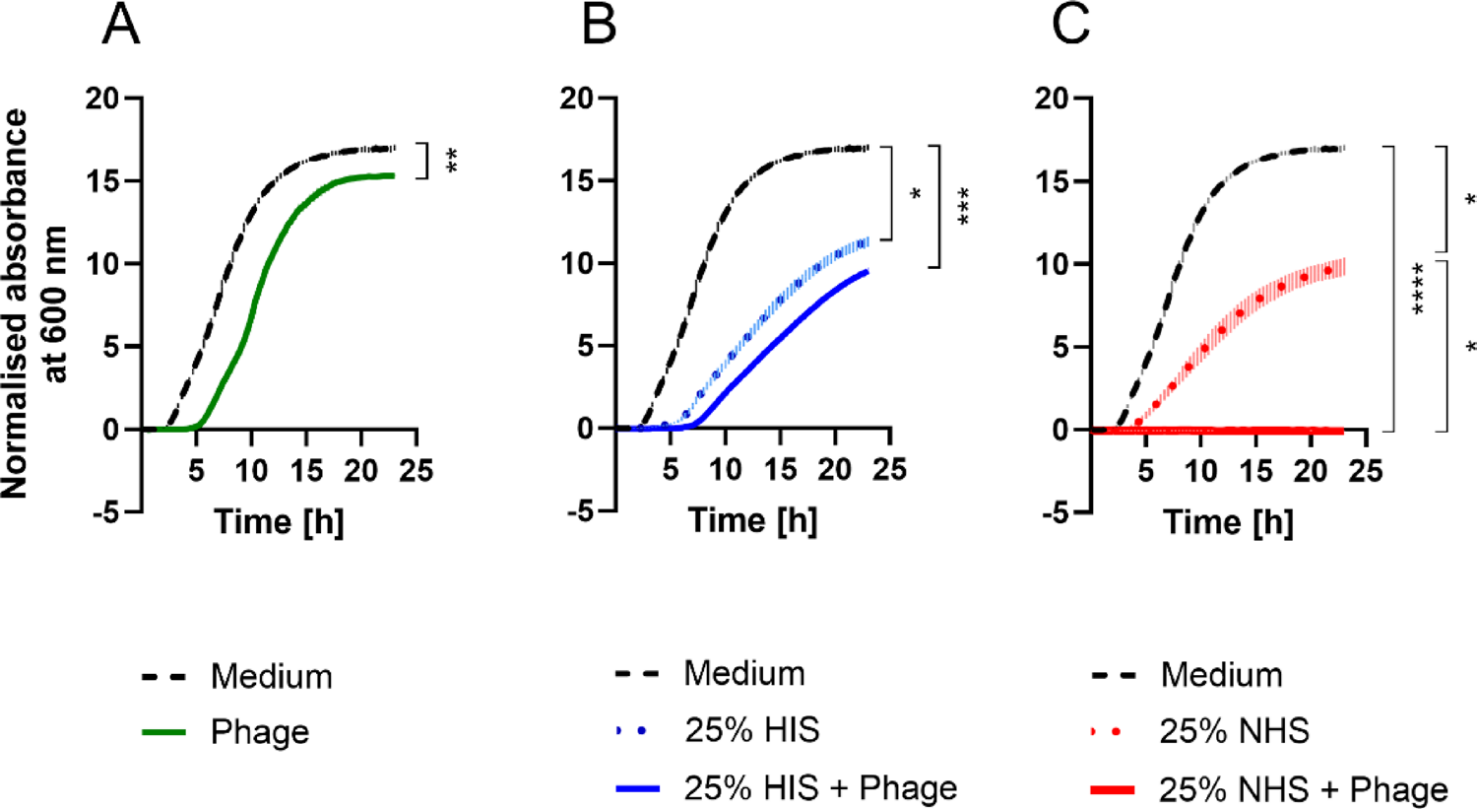
Synergistic effects of complement and bacteriophage fBenAci003 on killing *A. baumannii* 5910. Ten thousand cells of *A. baumannii* parental 5910 strain were seeded into each well and treated with fBenAci003 phage and or 25% serum. The bacterial growth was monitored by measuring absorbance at 600 nm. **A** Bacteria infected with the phage with a multiplicity of infection of 10 (MOI = 10). **B** Bacteria grown in 25% heat-inactivated serum (HIS) alone or in combination with the phage (MOI = 10). **C** Bacteria grown in 25% normal human serum (NHS) alone or in combination with the phage (MOI = 10). Plots of two independent biological experiments with technical triplicates. Means ± SEM of six measurements are shown (n = 6). The data were normalised to absorbance at 600 nm at t = 0 h, and the area under the curve (AUC) was calculated. To compare the AUC of two conditions, Mann–Whitney test was used (**A**). The AUC was analysed with ANOVA and Kruskal–Wallis test for multiple comparisons of *p*-values (**B**, **C**). *p < 0.0442, **p < 0.0022, ***p < 0.0002, ****p < 0.0001

According to in vivo studies, certain phages may become neutralised by C1q of the complement system or by preexisting antibodies in sera from mammals [32, 34, 60]. Hence, we initially assumed that the complement system might also neutralise the *Acinetobacter* phages, which would reduce the efficacy of the phages to infect their respective bacterial hosts. To examine whether the complement system inhibits phages from infecting and killing *A. baumannii* 5910, the bacterial growth was monitored when treated with the phage fBenAci003, heat-inactivated serum (HIS), normal human serum (NHS) or a combination of the phage and serum (Fig. 1). Upon treatment with the phage, the bacterial growth was delayed up to 5 h. Thereafter, the bacteria started to grow exponentially (Fig. 1A), indicating the development of resistance to the phage. Although the bacterial growth was more retarded upon treatment with 25% HIS or NHS compared to the phage only (Fig. 1B and C), the bacteria showed resistance to complement-mediated killing. Strikingly, when *A. baumannii* was incubated with NHS in combination with the phage, the bacterial growth was completely abolished (Fig. 1C). Importantly, we also observed the same phenomenon for other *A. baumannii* strains (Fig. S1). Together, this suggests a synergism between the complement system and phages in killing the tested *A. baumannii* strains.

### Phage-resistant bacteria are sensitive to complement

**Fig. 2:**
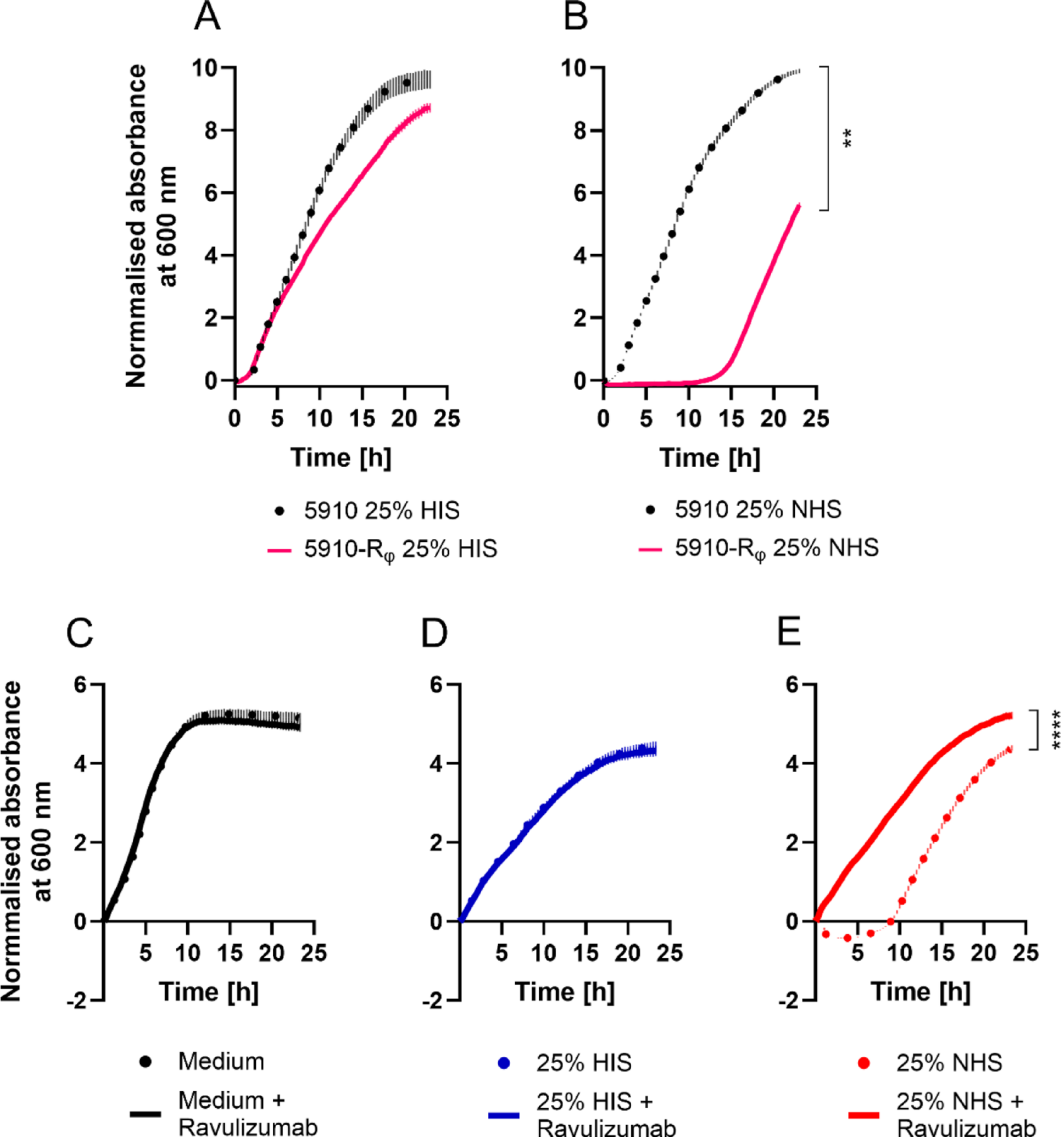
The phage-resistant 5910-R_φ_ clone is sensitive to killing by normal human serum. **A**, **B** Growth curves of the parental 5910 strain and the phage-resistant 5910-R_φ_ clone in serum. One million bacterial cells were seeded in each well with 25% HIS (**A**) or 25% NHS (**B**). Plots of two independent biological experiments with technical triplicates. Means ± SEM of six measurements are shown (n = 6). **C**–**E** Growth curves of phage-resistant *A. baumannii* 5910-R_φ_ in medium (**C**), 25% HIS (**D**), 25% NHS (**E**) and with or without the C5 inhibitor, ravulizumab (100 μg/ml). Ten million cells were seeded in each well. Depicted are plots of three independent biological experiments with technical triplicates. Means ± SEM of nine measurements are shown (n = 9). The data were normalised to absorbance at 600 nm at t = 0 h, and the AUC was calculated. To compare the AUC of conditions, Mann–Whitney test was used (A-E). **p < 0.0022, ****p < 0.0001

After observing the emergence of resistance to phage-mediated killing of *A. baumannii* 5910, a single phage-resistant clone (5910-R_φ_) was isolated. After verification of the phage resistance of 5910-R_φ_ (Fig. S2), these bacteria were treated with serum and the viability was monitored (Fig. 2). Remarkably, upon incubation with NHS, the bacterial growth of 5910-R_φ_ was delayed up to 13 h of incubation. This was in contrast to parental 5910 strain, which showed no growth reduction (Fig. 2B). Despite the 5910-R_φ_ clone showing less fitness in 25% NHS at early time points, these bacteria were able to survive in the serum environment as they began growing after 13 h of incubation. Interestingly, when the 5910-R_φ_ clone was incubated in NHS with a concentration below 4%, the growth of these bacteria was no longer hampered (Fig. S3). Indicating that the alternative pathway might be involved in the killing of the bacteria, since the alternative pathway usually is no longer active at serum concentrations below 5%.

To confirm that the 5910-R_φ_ clone was killed by the complement system, the monoclonal antibody ravulizumab was used to inhibit C5 to prevent the formation of membrane attack complex, C5b-9 [61]. Treatment of the 5910-R_φ_ clone with ravulizumab alone did not facilitate bacterial growth, as the growth kinetics were identical between the bacteria treated with medium or HIS only and when supplemented with ravulizumab (Fig. 2C and D). In contrast to the NHS-only treated 5910-R_φ_ clone, where the bacteria were rapidly lysed within the first 10 h, NHS supplemented with ravulizumab no longer killed the bacteria, as the monitored growth curve was similar to that of HIS-treated bacteria (Fig. 2E).

### Increased permeability and MAC deposition on phage-resistant *A. baumannii*

**Fig. 3:**
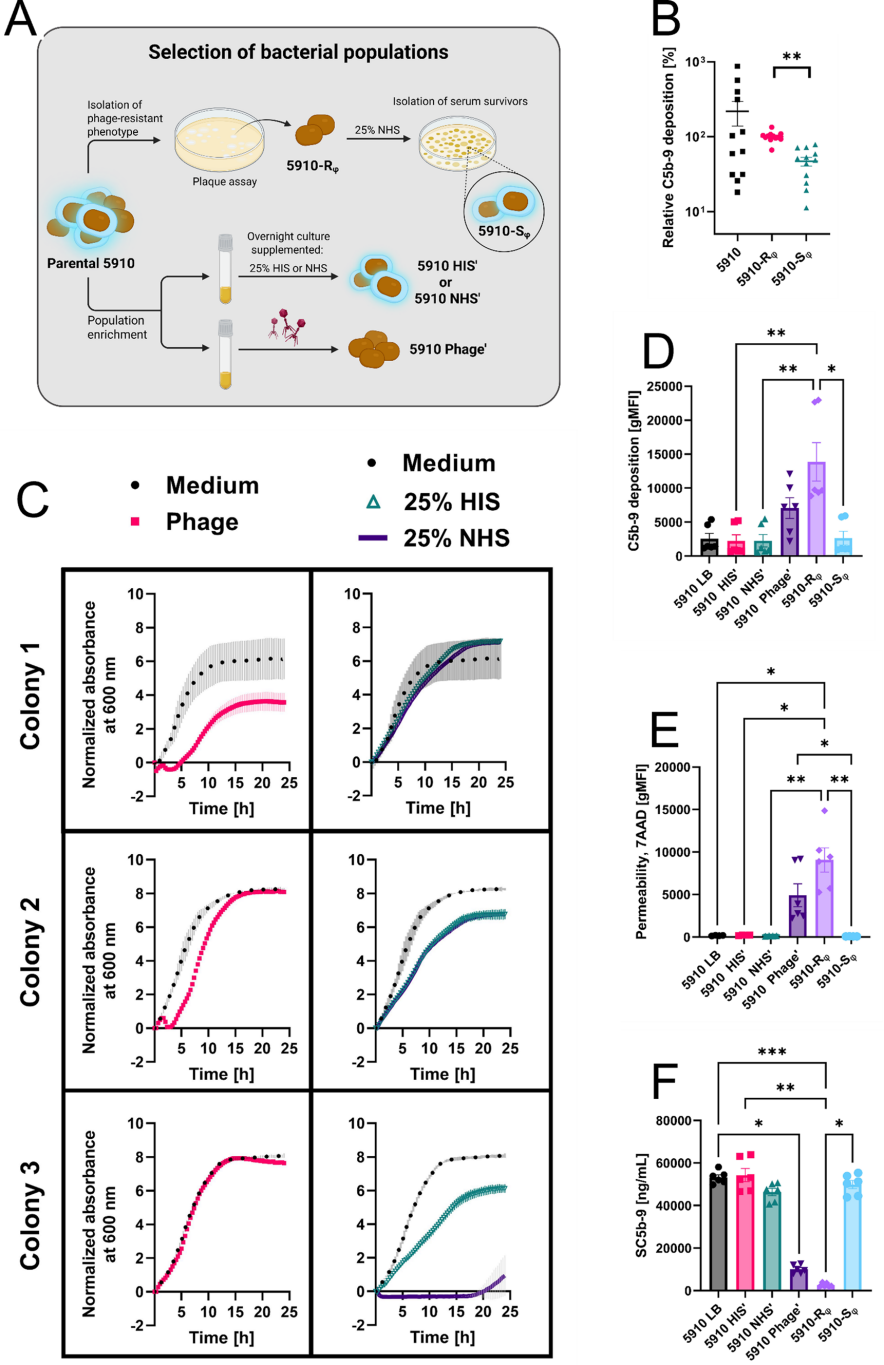
Phase variation in *A. baumannii* 5910 affects C5b-9 deposition on the bacterial surface. **A** Schematic illustration of bacterial population selection. To isolate the phage-resistant phenotype 5910-R_φ_, the parental 5910 strain was treated with the phage fBenAci003 through double-agar layer method, and a single colony grown on a clear plaque was isolated. 5910-R_φ_ was then treated with 25% NHS overnight, the survivors were plated and 5910-S_φ_ was isolated. To enrich a certain bacterial population, parental 5910 was preconditioned in liquid medium supplemented with 25% HIS (5910 HIS’), 25% NHS (5910 NHS’) or with the phage fBenAci003 (5910 Phage’) overnight. **B** C5b-9 deposition on the *A. baumannii* 5910 phenotypes. Parental 5910 strain, 5910-R_φ_, and 5910-S_φ_ were treated with 25% NHS for 30 min before staining with antibodies, and detection of C5b-9 deposition was measured with flow cytometry. To compare C5b-9 deposition between the different phenotypes from independent experiments, the gMFI was normalised according to the level of C5b-9 deposition on 5910-R_φ_ in each experiment. Six independent biological replicates with technical duplicates. Mean ± SEM of 12 measurements are shown (n = 12). **C** Phage- and serum sensitivity of different colonies of the parental *A. baumannii* 5910 strain. Ten million bacterial cells were seeded into each well and were either infected with the phage fBenAci003 (MOI = 0.1) or treated with 25% serum. The bacterial growth of three individual colonies of the parental 5910 strain was monitored by measuring the absorbance at 600 nm. The data were normalised to absorbance at 600 nm at t = 0 h. Means ± SD of technical duplicates are shown (n = 2). **D**, **E** C5b-9 deposition and outer membrane permeability on preconditioned *A. baumannii* 5910 phenotypes. The parental 5910 strain was preconditioned overnight with serum or with the phage fBenAci003 as depicted in Fig. 3A. All bacterial cultures were refreshed in LB without any supplements before treatment with 25% NHS for 30 min. The bacteria were stained with antibodies to detect C5b-9 deposition (**D**) and 7-AAD for permeability (**E**). 7-AAD is a membrane-impermeant DNA dye, which only stains cells with compromised membrane integrity. The signals were measured with flow cytometry. **F** Soluble C5b-9 formation on *A. baumannii* 5910 phenotypes. Bacteria were treated with 25% NHS for 30 min, and the amount of soluble C5b-9 formed in the supernatant was detected with ELISA. The level of complement autoactivation in serum at 37 °C over time is represented as NHS only condition without any bacteria. Data points represent three independent biological experiments with technical duplicates (**D**–**F**). Means ± SEM of six measurements are shown (n = 6). **B**, **D**–**F** Statistical analysis was performed with ANOVA and Kruskal–Wallis test for multiple comparisons of *p*-values. *p < 0.0350, **p < 0.0066, ***p < 0.0007

Upon prolonged incubation of the 5910-R_φ_ clone in NHS, a survivor clone (5910-S_φ_) was isolated (Fig. 3A). Interestingly, the 5910-S_φ_ clone showed similar features to the parental 5910 strain, as both strains were susceptible to the phage fBenAci003 and serum-resistant (Figs. S2 and S4). Following the above observations, we sought to investigate, whether the serum-sensitivity of the bacteria correlated with the level of permeability and the deposition of C5b-9 on the bacterial surface with flow cytometry. Indeed, the 5910-R_φ_ clone showed a 50% increase in C5b-9 deposition relative to 5910-S_φ_, while the parental 5910 strain showed a large variation (Fig. 3B). Heterogeneity in sensitivity to MAC attack among the parental 5910 strain indicated coexistence of different subpopulations, probably due to phase variation [62, 63]. Accordingly, we observed variations in phage- and serum sensitivity among the parental 5910 colonies (Figs. 3C, S5). Importantly, when the bacteria (colony 3) were resistant to the phage fBenAci003, they were sensitive to complement-mediated killing, and vice versa (colonies 1 and 2).

To enrich a certain subpopulation within a colony of the parental 5910 strain, the bacteria were preconditioned with 25% serum or with the phage fBenAci003 overnight (Fig. 3A). The enriched subpopulation was controlled by monitoring the bacterial growth in the presence of the phage fBenAci003 or with serum (Figs. S6–S7). Strikingly, all of the NHS preconditioned bacteria were sensitive to phage-mediated killing, while the bacteria that were preconditioned with phage were resistant (Figs. S6A, S7C and S7D). In contrast, LB or HIS preconditioned bacteria showed variation in phage- and serum-sensitivity between colonies (Fig. S6A, B). Thus, preconditioning of *A. baumannii* 5910 with NHS or phage could efficiently enrich a desired subpopulation. Nevertheless, after preconditioning with NHS (5910 NHS’), the bacteria showed a sixfold decrease in C5b-9 deposition compared to 5910-R_φ_ (Fig. 3D). Moreover, the membrane permeability of 5910-R_φ_ positively correlated with the level of C5b-9 deposition, where 5910-R_φ_ showed more than 70-fold increase compared to 5910 in LB (5910 LB), preconditioned with HIS (5910 HIS’), 5910 NHS’ or 5910-S_φ_ (Fig. 3E). Notably, soluble C5b-9 formation negatively correlated with the deposition of C5b-9 on the bacterial surface (Fig. 3F). This indicated that all *A. baumannii* phenotypes activated the complement system, but the C5b-9 complex was efficiently inserted only on the phage-resistant cells, which became thereby rapidly killed.

**Fig. 4:**
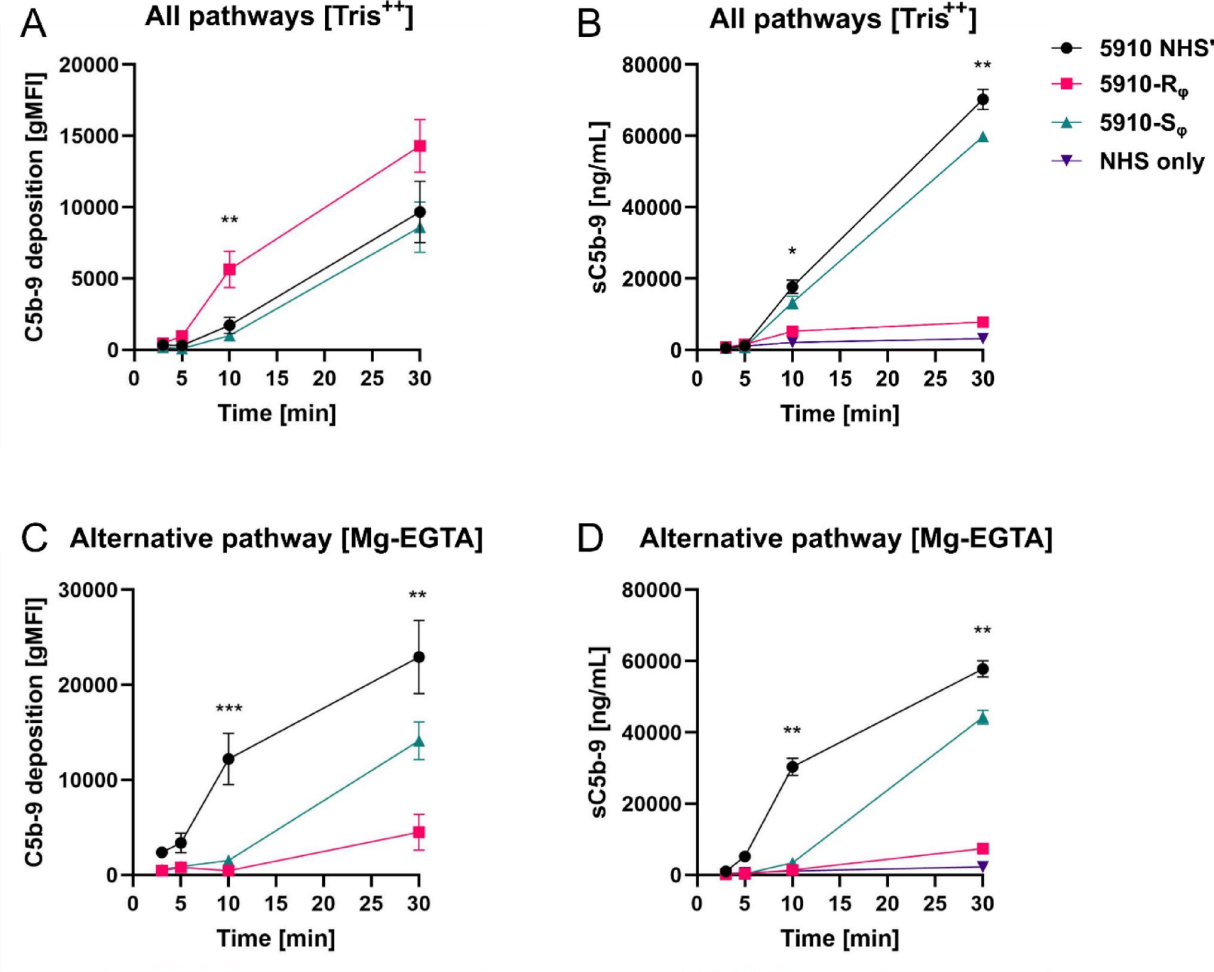
Kinetics of C5b-9 deposition on *A. baumannii* 5910 phenotypes. The parental 5910 strain preconditioned with 25% NHS (5910 NHS’), 5910-R_φ_ and 5910-S_φ_ were incubated in LB overnight. All bacterial cultures were refreshed in LB without any supplements before treatment with 25% NHS for 30 min. **A**, **B** The classical, lectin and the alternative pathways were activated when Tris.^++^ buffer was used. **C**, **D** The classical and lectin pathways were inhibited with Mg-EGTA. Activation of complement was measured at different time points by detecting C5b-9 deposition on the bacterial surfaces with flow cytometry (A,C) and the formation of soluble C5b-9 was determined by ELISA (B,D). The level of complement autoactivation in serum at 37 °C over time is represented as NHS only condition without any bacteria. Data points represent three independent biological replicates with technical duplicates (**A**–**D**). Means ± SEM of six measurements are shown (n = 6)

To gain a better understanding on the kinetics of C5b-9 formation by the different phenotypes, we chose to focus on the 5910 NHS’, 5910-R_φ_ and 5910-S_φ_ phenotypes for the measurement of the MAC deposition and formation of soluble C5b-9 over time (Fig. 4). When all the three complement pathways were active, C5b-9 was rapidly formed on the 5910-R_φ_ surface, and the deposition was significantly stronger compared to 5910 NHS’ and 5910-S_φ_ at 10 min of incubation with 25% NHS (Fig. 4A). In accordance with Fig. 3F, 5910 NHS’ and 5910-S_φ_ generated higher levels of soluble C5b-9 relative to 5910-R_φ_ over time (Fig. 4B). Probably, the detected level of soluble C5b-9 by 5910-R_φ_ came from autoactivation of the serum as indicated in the NHS only condition without any bacteria. Next, we inhibited activation of the classical and lectin pathways with Mg-EGTA to investigate whether the alternative pathway became activated by the different phenotypes. Interestingly, C5b-9 deposition on 5910-R_φ_ was retarded under alternative pathway activation conditions (Fig. 4C), whereas C5b-9 was rapidly deposited on the 5910 NHS’ at 10 min of incubation, and on the 5910-S_φ_ clone at a later time point. Finally, formation of soluble C5b-9 followed a similar trend as the deposition of C5b-9 on the bacteria, when only the alternative pathway was activated (Fig. 4D). Together, these results suggest that different complement pathways are activated depending on the bacterial phenotype. In the case of 5910-R_φ_, the classical and lectin pathways seem to play a crucial role in a rapid activation of the terminal pathway and killing of the bacteria, whereas the alternative pathway is predominantly activated by 5910 NHS’ and 5910-S_φ_. However, C5b-9 is rapidly shed from the 5910 NHS’ and 5910-S_φ_ clones to the environment.

### *Acinetobacter baumannii* undergoes morphological changes upon phage treatment

**Fig. 5:**
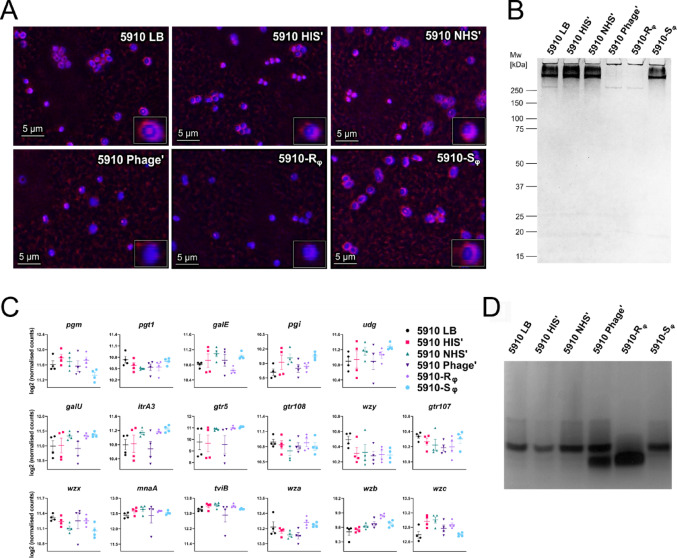
Phage treatment induces morphological changes in *A. baumannii* 5910. **A** Capsule staining of *A. baumannii* 5910 phenotypes**.** Parental 5910 strain was preconditioned with 25% HIS (5910 HIS’), 25% NHS (5910 NHS’) or with phage fBenAci003 (5910 Phage’), while 5910-R_φ_ and 5910-S_φ_ were grown in LB overnight. Bacterial cultures were pelleted and stained with India ink and 1% Crystal violet. **B** Capsular polysaccharide extraction. Capsular polysaccharides were isolated from overnight bacterial cultures and stained with 0.1% Alcian Blue after separation with SDS-PAGE. **C** Transcriptome analysis of the KL52 locus in *A. baumannii* 5910 phenotypes. Bacterial mRNA was isolated and sequenced. Plots of four biological replicates and mean ± SEM are shown (n = 4). Statistical analysis was performed with DESeq2 and Wald statistical test was used for comparison of means. **D** Analysis of extracted lipooligosaccharide (LOS). LOS were isolated from overnight cultures of the different *A. baumannii* 5910 phenotypes and silver stained after separation with SDS-PAGE. **A**, **B** and **D** are representative figures of two independent biological experiments

Next, we studied morphological changes of the bacteria after treatment with the phage fBenAci003 and serum. By staining the bacterial capsule and isolating capsular polysaccharides from the bacteria, the 5910 LB, 5910 HIS’, 5910 NHS’ and the 5910-S_φ_ phenotypes were found to produce an extensive amount of capsular polysaccharide, as these bacteria were positively stained with a halo around the cell. In contrast, the parental strain preconditioned with phage (5910 Phage’) and the 5910-R_φ_ phenotype lacked the capsule (Fig. 5A), and did not produce capsular polysaccharides with high molecular weight (Fig. 5B). To investigate which gene might be differentially expressed between the groups that resulted in the phenotypic change, we performed bulk RNA sequencing and analysed differentially expressed genes within the KL52 locus, which is responsible for capsule synthesis in the parental 5910 strain. Although the 5910 Phage’ and 5910-R_φ_ clones were non-encapsulated, we did not observe any genes in the KL52 locus that were differently expressed between the groups (Fig. 5C). Spike proteins of some phages can carry depolymerase activity, which degrades extracellular polysaccharides or capsular polysaccharides on the bacterial surface, facilitating the phages for the initial steps of host infection [64, 65]. Thus, the tail spike protein of phage fBenAci003 was analysed for depolymerase activity with the Phage Depolymerase Finder PhageDPO [58, 59]. Indeed, the phage tail spike protein was predicted to carry a depolymerase with a probability of 99% (Table S2). This suggested that the phage fBenAci003 degrades capsular polysaccharides of *A. baumannii* 5910 to initiate the infection.

Furthermore, we isolated lipooligosaccharides (LOS) from the bacteria and stained them with silver. In addition to the downregulation of capsular polysaccharide expression in 5910-R_φ_, these bacteria also synthesised shorter LOS relative to the parental 5910 strain preconditioned with serum and the 5910-S_φ_ clone, while 5910 Phage’ had both versions of LOS (Fig. 5D). The transcription levels of the genes within the outer core locus 1 (OCL1) that encodes enzymes catalysing the linkage of sugars to the outer core oligosaccharides of LOS and the genes involved in the inner core and lipidA synthesis were analysed. However, none of the genes were differently expressed between the groups (Fig. S8A). Moreover, we analysed the expression levels of genes involved in other lipopolysaccharide (LPS) or LOS biosynthesis pathways. Interestingly, lipopolysaccharide export system permease, *lptG*, was significantly downregulated in the 5910-R_φ_ clone (Fig. S8B). Altogether, the results indicate that the capsular polysaccharide and longer LOS protect *A. baumannii* from complement-mediated killing, but at least one of the two is essential for phage infection.

### Transposon mutagenesis downregulates capsule synthesis in *A. baumannii*

**Fig. 6:**
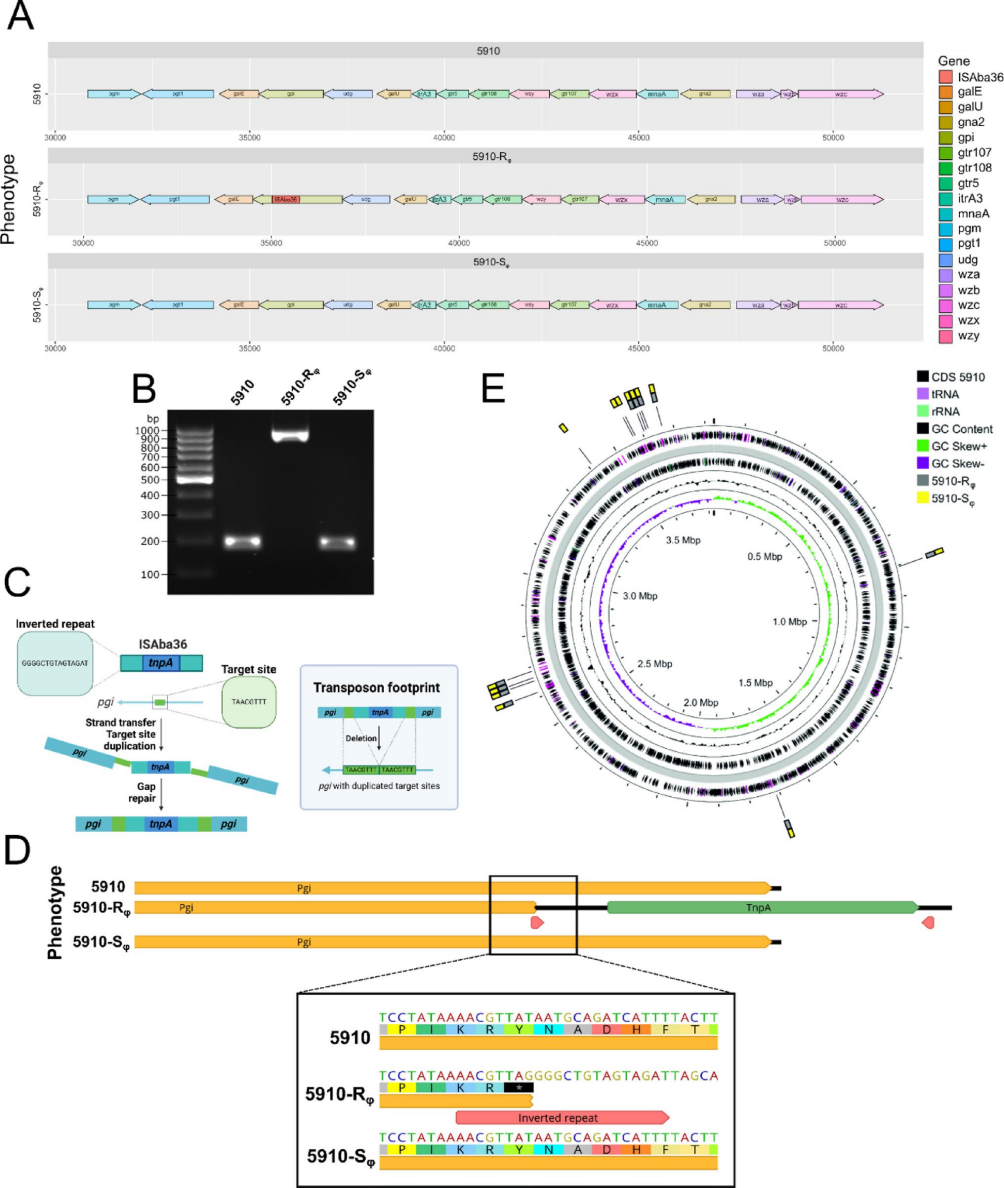
Transposon mutagenesis disrupts the KL52 locus in the *A. baumannii* phage-resistant 5910 phenotype. **A** Sequence alignment of KL52 locus between different phenotypes. Genomic DNA was isolated from overnight cultures of parental 5910 strain, 5910-R_φ_ and 5910-S_φ_. Sequenced genomes were assembled and the KL52 locus between the isolates was compared. Insertion sequence ISAba36 disrupting glucose-6-phosphate isomerase (*pgi*) in 5910-R_φ_ was identified by BLAST and ISFinder. **B** Verification of insertion sequence in *A. baumannii* 5910-R_φ_ clone. The local insertion region was amplified by PCR and separated in 2% agarose gel. The bands were extracted from the gel and sequenced. Representative gel picture of two independent biological replicates. **C** Schematic illustration of the mobile element, ISAba36, and transposon mutagenesis. Target site within *pgi* is duplicated upon integration of ISAba36 with its inherent transposase, TnpA. Upon deletion of the insertion sequence, duplicated target sites may remain as a footprint. **D** Insertion of ISAba36, generates a premature stop codon in *pgi* of the 5910-R_φ_ clone. Schematic illustration of the original coding sequence and the mutated Pgi, and transposase A (TnpA) in *A. baumannii* 5910 phenotypes. Red arrows: inverted repeats of the mobile element ISAba36. Black asterisk: stop codon. **E** Whole-genome of *A. baumannii* 5910 parental strain and the identified mutations in the 5910-R_φ_ and 5910-S_φ_ clones. The mutations in the clones were located relative to the parental 5910 strain by using variant calling tool on PATRIC. Grey box: mutations in 5910-R_φ_ clone. Yellow box: mutations in 5910-S_φ_ clone

Previously, it has been reported that *A. baumannii* undergoes spontaneous mutations upon treatment with phages [66, 67]. To address whether phage- treatment led to spontaneous mutations within the KL52 locus, the genomes of the parental 5910 strain, 5910-R_φ_ and 5910-S_φ_ clones were sequenced, and a comparison of the KL52 locus was performed. The sequence alignments between the parental 5910 and 5910-R_φ_ clones revealed that the gene glucose-6-phosphate isomerase (*pgi*) was disrupted with an insertion sequence in the non-encapsulated 5910-R_φ_ clone (Fig. 6A). The mutation within *pgi* was further confirmed by amplifying and sequencing the local area of the insertion site (Fig. 6B). The 712 bp insertion sequence was identified through BLAST as ISAba36 (accession number: MN735933.1), which is a transposon and encodes the transposase TnpA. Similarly, ISAba36 was also obtained from the ISfinder database (accession number: KU667287) and BLASTn sequence alignment showed 99% identity. Additionally, ISFinder identified TnpA to be a DDE-type transposase belonging to IS family IS1595 and the group IS1016. Flanking the *tnp*A gene, there were inverted repeats (IRs) with a sequence GGGGCTGTAGTAGAT (Fig. 6C). This sequence contains the consensus sequence GGGgctg found in IRs of IS1016 as reported in an earlier study [68]. Remarkably, the integration of ISAba36 in *pgi* in the 5910-R_φ_ sequence created a premature stop codon between the direct repeat and the left IR of ISAba36 (Fig. 6D). This suggests that downregulation of capsular polysaccharide expression could occur at the translational level of *pgi*.

Upon insertion of the DDE transposon into the target site and repair of the broken DNA strands, direct repeats were formed on both sides of the integrated transposon (Fig. 6C). Generation of direct repeats can be useful for tracking past transposon mobility events, as they remain as a footprint in the excision site after the transposon has been removed [69, 70]. As illustrated in Fig. 6C, direct repeats were found in the 5910-R_φ_ sequence flanking the integrated ISAba36, with a sequence, TAACGTTT. Upon transposon excision, the direct repeats will often remain at the site, however, such duplication was not found in the *pgi* of 5910-S_φ_ sequence. Indicating that 5910-R_φ_ could revert to its original encapsulated phenotype through a scarless DNA repair mechanism when challenged with NHS. Importantly, although *pgi* remains intact in 5910-S_φ_, this clone carried mutations which were commonly found in 5910-R_φ_, but not in the parental 5910 strain (Fig. 6E, Table S3), ruling out possible contamination from the parental 5910 strain.

## Discussion

In this study we observed that the combination of complement-preserved serum and phages totally abrogated the viability of different *A. baumannii* strains, reflecting a synergistic effect (Figs. 1C and S1C). Initially, we hypothesised that the complement system may inhibit the phages from killing the bacteria, which has been demonstrated in a previous study showing that *P. aeruginosa* -specific phages having myovirus morphology were inhibited by C1q [34]. However, in the same work, four phages having podovirus morphology were found to be insensitive to complement-mediated inhibition. The three phages used in this work, fBenAci001, fBenAci002 and fBenAci003, belong to the *Autographiviridae* family [37] and are structurally podoviruses, which encouraged us to hypothesise that these phages would tolerate complement. Our promising results call for additional work regarding the effect of complement on phages representing different morphological and taxonomical groups. This work is needed for understanding the overall picture in phage-complement interactions, especially because of the potential therapeutic implications they may have during the era of progressively increasing problems related to antimicrobial resistance.

Like many *Acinetobacter* spp., *A. baumannii* 5910 was resistant to complement-mediated killing and able to grow in 25% NHS (Fig. 1B and C). Although the parental 5910 strain was initially sensitive to phage fBenAci003, the strain rapidly adapted and became resistant to the phage in standard culture conditions (Fig. 1A). Interestingly, the phage-resistant 5910-R_φ_ clone was rapidly killed by NHS (Fig. 2B), which concurs with previous reports on other *A. baumannii* phages [67, 71]. Although complement is inactivated in HIS, the growth curves of the parental 5910 strain and 5910-R_φ_ were retarded in 25% HIS condition relative to the medium control (Figs. 1B, 2C and D). Possibly, both phenotypes were sensitive to heat-stable proteins in serum, such as antimicrobial peptides, antibodies or C1q [72, 73]. Additionally, we verified the role of complement in clearing 5910-R_φ_ by the ability of ravulizumab (anti-C5 antibody) to prevent bacterial lysis (Fig. 2E). The 5910-R_φ_ clone also showed a higher level of C5b-9 deposition and an increased permeability (Fig. 3D and E). Altogether, the results show that the complement system is activated and kills the phage-resistant *A. baumannii* phenotype.

Furthermore, we extended previous findings of phage-resistant *A. baumannii* becoming resensitised to serum complement by characterising the complement pathways that play a role in killing the different *A. baumannii* phenotypes. When all the three complement pathways were active, C5b-9 was more extensively formed on 5910-R_φ_ at the 10 min time-point relative to 5910 NHS’ and 5910-S_φ_ (Fig. 4A). In contrast, when the classical and lectin pathways were inhibited, 5910 NHS’ and 5910-S_φ_ showed a higher level of C5b-9 deposition over time compared to 5910-R_φ_ (Fig. 4C). Importantly, 5910 NHS’ and 5910-S_φ_ induced high levels of soluble C5b-9 even when the classical and lectin pathways were inhibited (Fig. 4B and D). This indicated that the 5910 NHS’ and 5910-S_φ_ clone can escape the complement system by shedding C5b-9 from the bacterial surface. Although these observations strongly suggest that the classical and lectin pathway are important for the rapid generation of C5b-9 on 5910-R_φ_, we do not exclude the importance of the alternative pathway in killing this clone. When the 5910-R_φ_ clone was cultivated at serum concentrations below 4%, the bacteria grew as well as under serum-free conditions (Fig. S3). This suggests that the alternative complement pathway is involved in the killing of 5910-R_φ_, since at concentrations below 5% the activity of the alternative pathway becomes limited [74, 75]. Possibly, the classical and lectin pathways are crucial for initial activation of the complement system, while the alternative pathway is necessary for amplifying the activation and further promoting activation of the terminal pathway. Notably, in a real-life situation complement activation would also boost phagocytosis by attracting and activating neutrophils to attack complement-opsonised bacteria.

According to previous studies, *Acinetobacter* capsule is an important complement escape mechanism by shielding the outer membrane from C5b-9 deposition [17, 21, 23, 24]. In line with earlier findings [67, 76], the 5910 Phage’ and 5910-R_φ_ phenotypes had indeed downregulated capsule expression (Fig. 5A and B). Moreover, after exposure to the fBenAci003 phage, the length of the LOS of these bacteria became shorter compared to the 5910 LB, 5910 HIS’, 5910 NHS’ and 5910-S_φ_ phenotypes (Fig. 5D). Notably, the expression level of capsular polysaccharides and longer LOS in 5910 NHS’ and 5910-S_φ_ also reflected their ability to activate the alternative pathway more rapidly than 5910-R_φ_ (Fig. 4C), as demonstrated in other bacterial species [77, 78]. This morphological change had been observed previously [76, 79], and suggests that longer LOS in 5910 might serve as a receptor for fBenAci003 to infect its host. In any case, the longer LOS might be advantageous for 5910 to evade the complement system, as the presence of longer LPS in *A. baumannii*, *Salmonella* spp, *Coxiella burnetii*, and *Klebsiella pneumoniae* protects these bacteria from complement-mediated killing [22, 80–83]. Certainly, further studies are required to estimate the relative contributions of the capsular polysaccharides and LOS in complement evasion and phage fBenAci003 sensitivity of *A. baumannii*.

Recently, it has been reported that phage-resistant *P. aeruginosa* with a defect in the LPS synthesis machinery, generated lower levels of soluble C5b-9 and was susceptible to killing by serum complement [84]. A similar phenomenon was also observed in our study (Figs. 3F and 5D). Additionally, we demonstrated that a shorter LOS correlated with the more abundant deposition of C5b-9 and membrane permeability (Figs. 3D, E and 5D). Notably, the 5910 Phage’ had longer and shorter LOS, but more C5b-9 deposited to the 5910-R_φ_ clone, which only synthesised shorter LOS. Altogether our results indicate that the complement system is activated by all *A. baumannii* phenotypes. However, the expression of capsular polysaccharides and longer LOS prevented efficient insertion of the C5b-9 complex on the bacterial outer membrane. Instead of membrane insertion, the C5b-9 complex became shed to the environment.

Interestingly, the tail spike proteins of the *Acinetobacter* phages often carry depolymerase activity, which can cleave capsular polysaccharides. In previous studies, treatment of capsular polysaccharides of the targeted hosts with purified depolymerases from the *A. baumannii* phages led to serum sensitisation [85–87]. Given that the spike protein of fBenAci003 phage has been predicted to have depolymerase activity (Table S2) and none of the genes in the K locus were significantly differentially expressed at the transcriptomic level (Fig. 5C), the phage depolymerase might potentially have degraded the capsular polysaccharide, making these bacteria become sensitive to complement. However, the quantity of the enzyme in assays using purified depolymerase is in general higher and the degradation of the bacterial capsule more efficient compared to the depolymerase that is part of the phage tail. Moreover, the MOIs that were used in this study were relatively low, suggesting that the phages probably used the depolymerase to recognise the capsular polysaccharide as a primarily receptor, and their concentration may not have been sufficient enough to completely degrade the capsule during the infection of the host.

To completely avoid phage attack, the bacteria became resistant to the phage by downregulating capsule synthesis through transposon mutagenesis. As a response to selection pressure, the chromosomal DNA of *A. baumannii* can undergo spontaneous mutations [66, 67, 71, 76, 88]. Disruption of the K locus through the insertion of transposases, IS4 and IS5, has previously been observed in phage-resistant *A. baumannii* strains [76]. In our study, we report transposon mutagenesis as a phage-resistance mechanism in *A. baumannii* 5910, where the insertion of ISAba36 at *pgi* could lead to the downregulation of capsule expression (Figs. 5, 6A and B). Although the *pgi* mRNA expression level was not differentially expressed between the phenotypes, a premature stop codon was observed in *pgi* upon integration of ISAba36 (Figs. 5C and 6D). This indicates that the mutation leads to a truncated Pgi without the Lys519 residue, which is one of the important active sites in the C-terminal part of Pgi [89] (UniProt accession ID: B0VMS2). Thus, truncation of Pgi most probably reduced or completely abolished its enzymatic activity, thereby leading to downregulation of capsule synthesis.

Previous studies have shown that genes at the outer core locus is often downregulated or mutated in *A. baumannii* after phage treatment, which potentially could result to the production of truncated LOS [76, 90]. Despite the fact that phage treatment led to truncated LOS production in *A. baumannii* 5910, we did not observe any mutations nor downregulation of transcription of genes within the OCL1 locus (Fig. S8A). Given that depolymerases can also degrade LPS [91, 92], we do not exclude the possibility that fBenAci003 might have cleaved the LOS of the host. Certainly, changes in other LPS or LOS biosynthesis pathways could also affect the LOS composition. Among these pathways, the Lpt machinery is important for transportation of LPS to the outer membrane, where LptF and LptG are responsible for the extraction of LPS from the bacterial inner membrane and delivery of the LPS molecule to Lpt proteins in the periplasm and outer membrane [93, 94]. Indeed, the 5910-R_φ_ clone significantly downregulated the transcription of *lptG*, but we did not observe a similar trend in the 5910 Phage’ phenotype (Fig. S8B). Possibly, truncation of LOS in the 5910 Phage’ phenotype is due to downregulation of genes in other biosynthesis pathways, or degradation by phage depolymerase. Furthermore, we confirmed that *lptG* was not mutated in the 5910-R_φ_ clone. Hence, the relation of *lptG* and the functional outcome of LOS composition in *A. baumannii* needs to be assessed through additional studies in the future. As Pgi catalyses the interconversion of glucose-6-phosphate to fructose-6-phosphate, we speculate that the mutation of this enzyme might indirectly affect the synthesis of LOS in *A. baumannii*, by causing an imbalance in the carbohydrate metabolism or in the production of substrates for LOS synthesis. In line with previous observations [76, 90], downregulation of both capsular polysaccharides and LOS seems to be essential for the fBenAci003 phage to recognise *A. baumannii* 5910. However, further studies are required to confirm whether the loss of capsule or longer LOS affects the adsorption of phages to the host and pinpoint which serves as a primary receptor for fBenAci003 phage.

Transposon insertion typically leads to target-site duplication, flanking the termini of the transposon. Upon excision of the transposon, target-site duplication often remains as a footprint, which can be used for tracking transposon activities [69, 70]. However, seamless repair of the DNA after the excision of the transposon has also been reported, but most of these observations have been made with eukaryotic transposons [69, 70]. To the best of our knowledge, homologous recombination and the RecBCD complex have been reported as repair mechanisms in prokaryotes during transposon transfer to the target DNA [69, 70], while seamless DNA repair mechanisms after transposon excision have been less intensely studied. Recently, seamless DNA repair has been reported in *A. baumannii*, showing that capsule production can be restored after prolonged exposure to antibiotics [95]. Similarly, our study demonstrated that the non-encapsulated phenotype could revert to an encapsulated phenotype after serum exposure through the excision of the mobile element ISAba36 from *pgi* (Fig. 6). To eliminate the possibility that 5910-S_φ_ is contaminated with the parental 5910 strain, 5910-S_φ_ were compared with 5910-R_φ_ and parental 5910 for mutations that were commonly shared. Indeed, 5910-S_φ_ carried mutations that were found in the 5910-R_φ_ clone, but not in the parental 5910 strain (Fig. 6E and Table S3). This supported the possibility that 5910-S_φ_ might have vertically inherited these mutations from the 5910-R_φ_ clone, and the DNA was repaired seamlessly after transposon excision from *pgi*. Together with the observations of shared similar features between the parental 5910 strain and the 5910-S_φ_ clone (Figs. S2, S4 and 5), our findings indicate that transposon mutagenesis is a reversible process that can be induced through prolonged serum exposure, in which *A. baumannii* 5910 can adapt to phage or NHS conditions.

On the other hand, the absence of target-site duplication at *pgi* in the 5910-S_φ_ clone supports the idea of the existence of heterogenous subpopulations and that the selection pressure can skew the distribution towards a predominant population with a distinct phenotype. Phase variation has been studied in *A. baumannii* and contributes to population diversity, where the subpopulations exhibit different morphologies and virulence [96–99]. Nevertheless, our observation of large variation of C5b-9 deposition and viability in serum of the parental 5910 strain also reflects heterogeneity (Fig. 3B and C). By means of preconditioning the parental 5910 strain with NHS or with the fBenAci003 phage, their viability in serum and C5b-9 deposition were less variable, apparently as a result of homogeneous population enrichment (Figs. 3D, S6 and S7).

Currently, case studies have reported the use of phages to treat infections caused by multidrug-resistant *A. baumannii*. In certain instances, *A. baumannii* infections have been successfully cleared [100–104]. Notably, during the time course of phage therapy of a patient with necrotising pancreatitis complicated by multidrug-resistant *A. baumannii*, the bacteria developed resistance to the phages that were administered to the patient. Further characterisation of the phage-resistant isolates showed a gradual morphological change towards a non-encapsulated phenotype which carried mutated *gtr76* within the KL116 locus [66, 100]. We observed a similar phenomenon in vitro, when the 5910 strain became resistant to phages due to transposon mutagenesis in *pgi* (Figs. S2, 5 and 6). Thus, with the concern for phenotypic variations in *A. baumannii*, we emphasise the importance of preconditioning clinical isolates of bacteria in a way that mimics in vivo settings. This would be relevant during the development of alternative therapeutic means and for evaluating conventional and combinatorial therapy outcomes. These conditions should be expanded to study the efficacy of various antibiotics and the sensitisation of bacteria under phage and complement selection. In this regard, our study highlights the synergy between phages and the complement system in clearing *A. baumannii*. Importantly, the synergism between the complement system and phages in killing the tested *A. baumannii* strains, as observed in our study, may also indicate a preserved bactericidal effect against many other *A. baumannii* strains. Knowing that the phages carry depolymerase activity (Table S2), suggests that they can degrade the capsular polysaccharide on the bacterial surface, which allows the complement C5b-9 complex to be deposited more readily on the outer membrane.

During an infection and tissue damage, complement becomes activated and can become depleted from the area of infection. Also, there may not be enough complement in wounds, body cavities or other areas. Therefore, supplying these areas with fresh complement together with phages could be vital for clearing an infection caused by multidrug-resistant bacteria. Furthermore, supplying the areas with anti-bacterial antibodies simultaneously—either separately as passive antibodies or in immune serum with complement—would boost the killing efficacy of complement. Using endogenous patient serum could be effective, especially when the bacteria exist in their non-encapsulated phenotype as a predominant subpopulation. Studies have shown that the use of antibodies could be a potential therapy to treat infections caused by *A. baumannii* [105–107]. However, due to the diversity of capsular polysaccharides in *A. baumannii*, the development of capsule-targeting monoclonal antibodies for therapeutic use would be challenging [108, 109]. On the other hand, the conserved outer membrane protein, OmpA, is considered as a more appropriate vaccine candidate [110], though, capsular polysaccharides can prevent antibodies from binding to OmpA leading to a reduced efficacy [109]. We envision that immunotherapy can be boosted by phages that degrade capsular polysaccharides, exposing OmpA or other targets to immune serum containing both anti-bacterial antibodies and complement.

## Supplementary Information

Below is the link to the electronic supplementary material.

Supplementary Material 1

## Data Availability

For the whole genome and RNAseq data, all can be retrieved through BioProject: PRJNA1074513 in GenBank. The raw data will be publically available once the manuscript is accepted. For the moment, the raw data can be reviewed through the following link: https://data.mendeley.com/preview/z59ccd4dp5?a=7333d928-4824-4ac5-8e48-ca447f68b62f.
