# Supplementary material for: Antibiotic-resistant Acinetobacter baumannii can be killed by a combination of bacteriophages and complement
Source: Med Microbiol Immunol. 2025 Sep 2;214(1):40. doi: 10.1007/s00430-025-00852-0 (PMC12405337; doi:10.1007/s00430-025-00852-0)
Supplement: Supplementary file 1 — Supplementary Material 1 [file 430_2025_852_MOESM1_ESM.pdf]

Supplementary materials

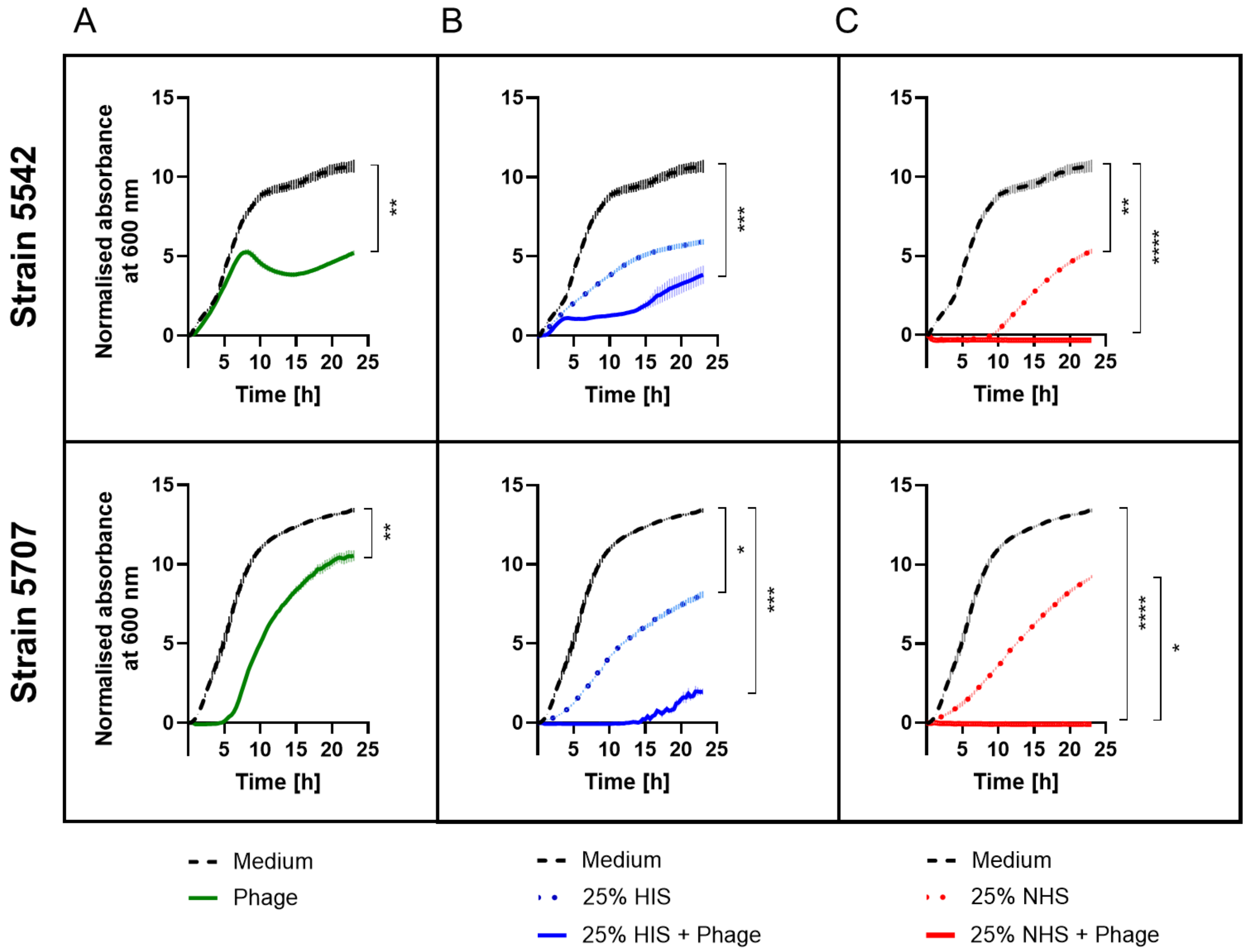

Fig. S1: A combination of complement and bacteriophages kills *A. baumannii* strains. One million cells of *A. baumannii* 5542 or 5707 were seeded into each well and treated with the phage and or with 25% serum. The bacterial growth was monitored by measuring absorbance at 600 nm. **A** The strain 5542 was infected with phage fBenAci001 (MOI = 5) and the strain 5707 with phage fBenAci002 (MOI = 0.01). **B** Bacteria grown in 25% heat-inactivated serum (HIS) alone or in combination with a phage (fBenAci001 MOI = 5, fBenAci002 MOI = 0.01). **C** Bacteria grown in 25% normal human serum (NHS) alone or in combination with a phage (fBenAci001 MOI = 5, fBenAci002 MOI = 0.01). Plot of two independent biological replicates with technical triplicates. Means ± SEM of six measurements are shown (n = 6). Data were normalised to absorbance at 600 nm at t = 0 h, and the area under the curve (AUC) was calculated. To compare the AUC of two conditions, Mann-Whitney test was used (A). The AUC was analysed with ANOVA and Kruskal-Wallis test for multiple comparisons of p-values (B,C). *p<0.0235, **p<0.0043, ***p<0.0006, ****p<0.0001.

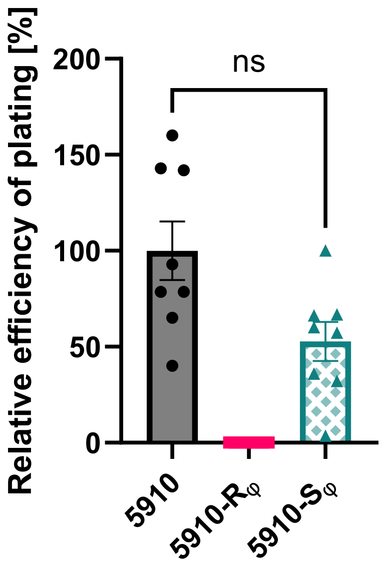

Fig. S2: Phage infectivity of *A. baumannii* 5910 phenotypes. Relative efficiency of plating (EOP) was calculated relatively to the number of plaques that were observed on the parental 5910 strain that was infected with the fBenAci003 phage. No plaques were observed in the phage-resistant isolate (5910-R_{φ}). Phage infectivity was also tested for the 5910-S_{φ} clone, which is an isolate of 5910-R_{φ} that survived in 25% NHS. Each data point represents the EOP of at least two technical replicates of three independent experiments. Means ± SEM of three biological experiments with at least two technical replicates (n = 8) are shown. Statistical analysis was performed with ANOVA and Kruskal-Wallis test for multiple comparisons of p-values. ns: non-significant.

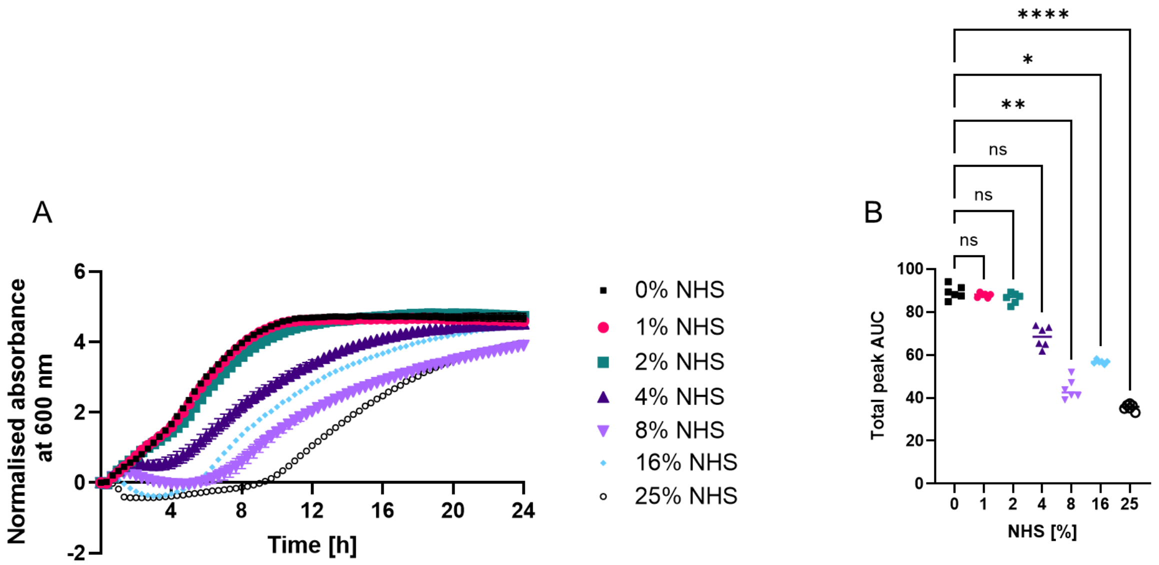

Fig. S3: Growth curves of phage-resistant *A. baumannii* 5910-R$_{\varphi}$ in sublytic serum concentrations. Ten million cells of the phage-resistant *A. baumannii* 5910-R$_{\varphi}$ phenotype were seeded into each well and were grown in various concentrations of normal human serum (NHS). **A** The bacterial growth was monitored by measuring the absorbance at 600 nm. Plots of three independent biological experiments with technical duplicates. Means ± SEM of six measurements are shown (n = 6). Data were normalised to absorbance at 600 nm at t = 0 h, and the area under the curve (AUC) was calculated. **B** The AUC was analysed with ANOVA and Kruskal-Wallis test for multiple comparisons of p-values. *p<0.0265, **p<0.0013, ****p<0.0001, ns: non-significant.

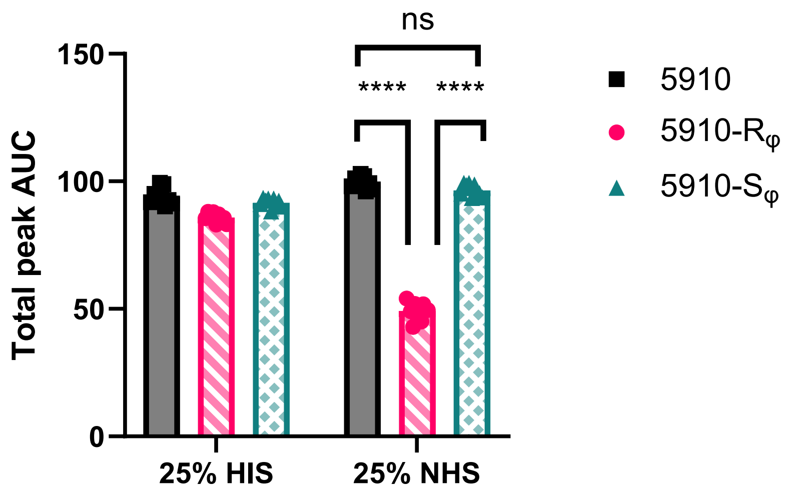

Fig. S4: Viability of *A. baumannii* 5910 phenotypes in normal human serum. Ten million cells of parental 5910 stain, 5910-$R_φ$ and 5910-$S_φ$ clones were seeded into each well and grown in 25% heat-inactivated serum (HIS) or normal human serum (NHS). Bacterial growth curves were monitored for 24 hours. Total peak areas under the curves (AUC) were calculated from three independent biological experiments with technical triplicates. Means ± SEM of three biological experiments with technical triplicates (n = 9) are shown. Statistical analysis was performed with ANOVA and Kruskal-Wallis test for multiple comparisons of p-values. ****p< 0.0001, ns: non-significant.

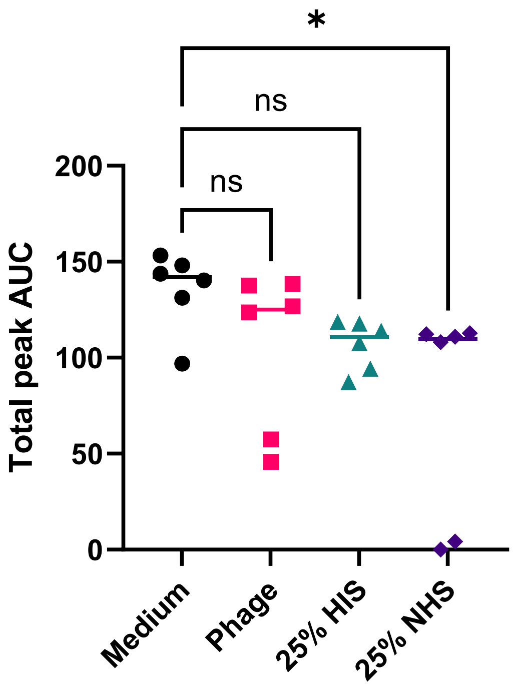

Figure S5: Statistical analysis of bacterial growth curves of parental *A. baummannii* 5910 in different conditions. The phage- and serum-sensitivity of different colonies of the parental *A. baumannii* 5910 strain was evaluated by measuring the absorbance at 600 nm. The AUC were calculated from Figure 3C. Plot of three individual colonies with technical duplicates. Means ± SEM of six measurements are shown (n = 6). Statistical analysis was performed with ANOVA and Kruskal-Wallis test for multiple comparisons of p-values. *p<0.0128, ns: non-significant.

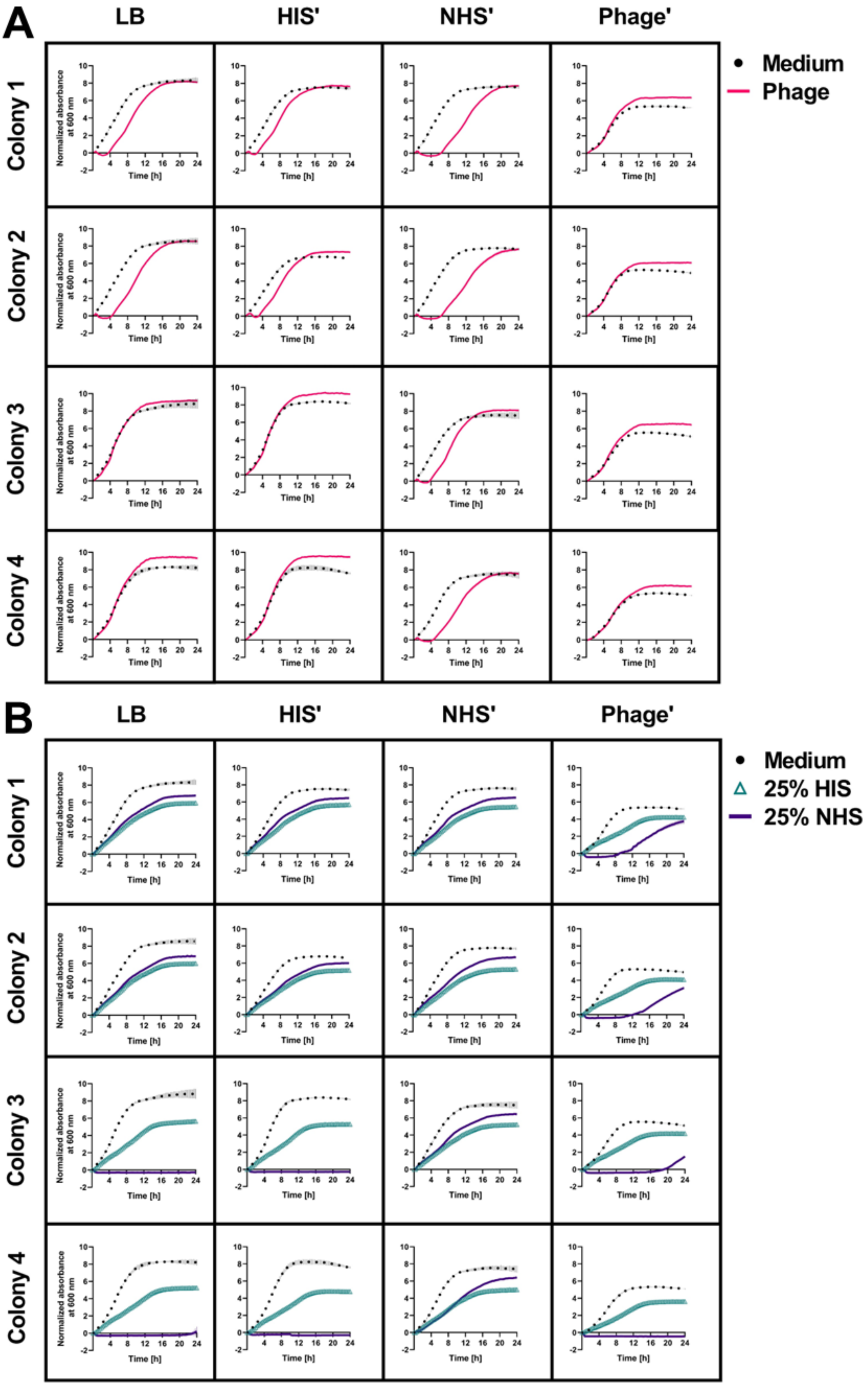

Fig. S6: Growth curves of the preconditioned parental *A. baumannii* 5910 strain. The parental 5910 strain was preconditioned with 25% HIS (HIS'), 25% NHS (NHS') or the phage

fBenAci003 (Phage') overnight and refreshed in LB medium without any supplements before seeding ten million bacterial cells into each well. **A** The bacteria were infected with the phage fBenAci003 (MOI = 0.1), **B** or treated with 25% serum. The bacterial growth was monitored by measuring absorbance at 600 nm. Means ± SD of technical duplicates (n = 2) are shown. The data were normalised to absorbance at 600 nm at t = 0 h.

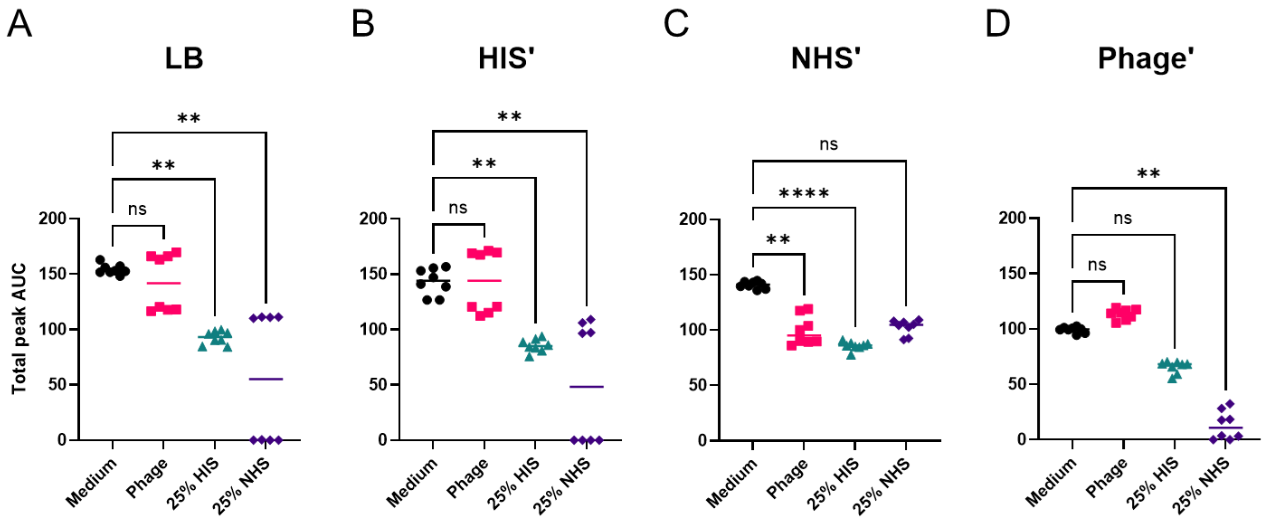

Fig. S7: Statistical analysis of the growth curves of the preconditioned parental *A. baumannii* 5910 strain. The parental 5910 strain was cultivated in LB (**A**), preconditioned with 25% HIS (HIS', **B**), 25% NHS (NHS', **C**) or the phage fBenAci003 (Phage', **D**) overnight and refreshed in LB medium without any supplements before seeding ten million bacterial cells into each well. The bacteria were infected with the phage fBenAci003 (MOI = 0.1), or treated with 25% serum. The bacterial growth was monitored by measuring absorbance at 600 nm. The data were normalised to absorbance at 600 nm at t = 0 h, and the AUC was calculated from Figure S6. Plot of four individual colonies with technical duplicates. Means ± SEM of eight measurements are shown (n = 8). Statistical analysis was performed with ANOVA and Kruskal-Wallis test for multiple comparisons of p-values. **p<0.0071, ****p< 0.0001, ns: non-significant.

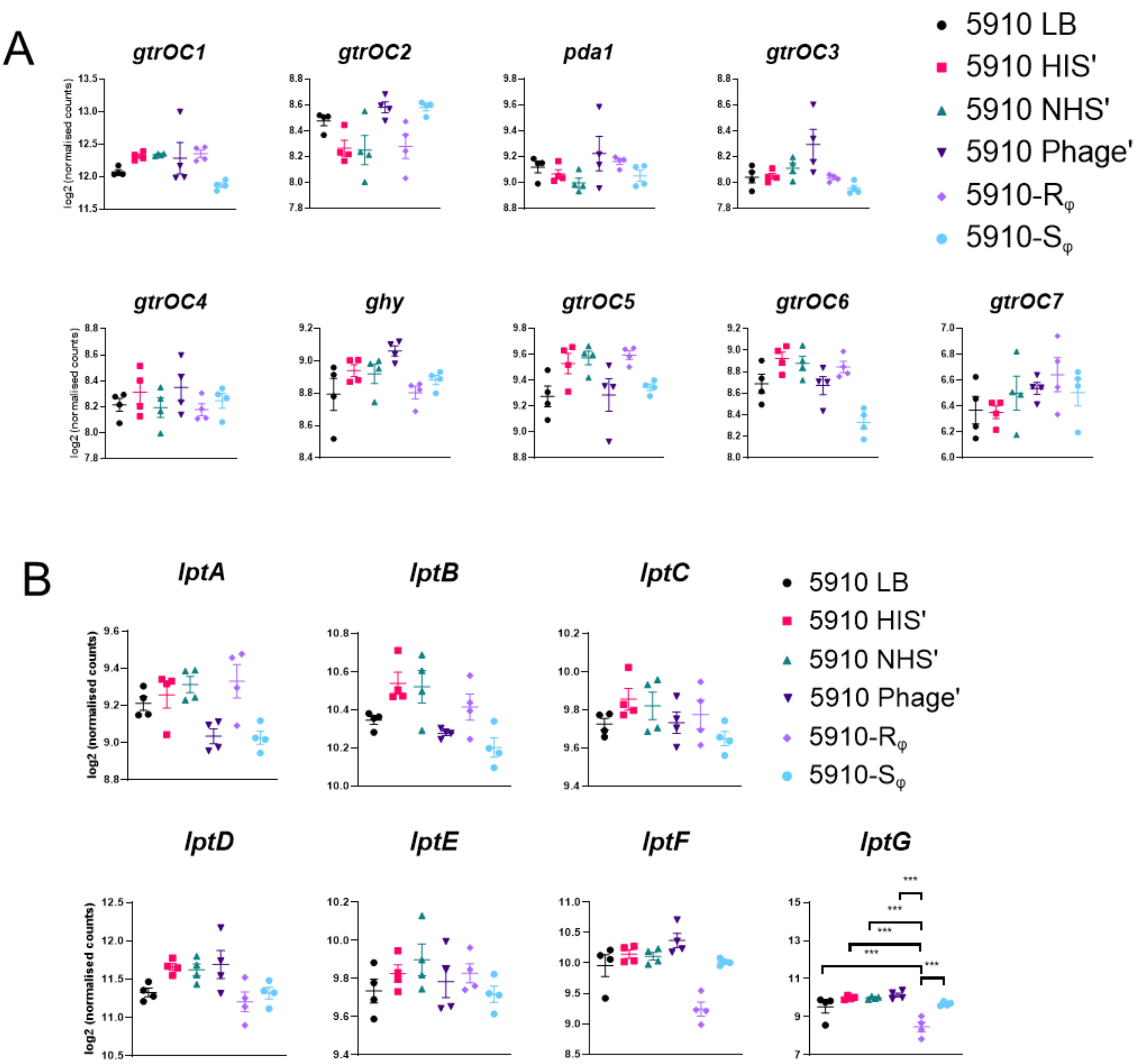

Fig. S8: Transcriptome profile of OCL1 locus and lipopolysaccharide transport (Lpt) machinery in *A. baumannii* 5910 phenotypes. Parental 5910 strain was preconditioned with 25% HIS (5910 HIS'), 25% NHS (5910 NHS') or with the phage fBenAci003 (5910 Phage'), while 5910-R$_{\varphi}$ and 5910-S$_{\varphi}$ were grown in LB overnight. Bacterial RNA was isolated and sequenced. Statistical analysis of the differentially expressed genes in the OCL1 locus (**A**) and the Lpt machinery (**B**) was performed with DESeq2 and Wald statistical test was used for comparison of means. Plots of four independent biological replicates and means ± SEM are shown (n = 4).

Table S1: *Acinetobacter baumannii* phenotypes used in this study. 5910-R_φ phenotype was isolated from a plaque after treating the 5910 parental strain with fBenAci003 phage. 5910-R_φ was subsequently treated with 25% NHS, and the survivor 5910-S_φ was obtained. To enrich a certain subpopulation within the parental 5910 strain, a colony was either inoculated in LB supplemented with 25% HIS, 25% NHS or 10 µl of phage fBenAci003 lysates (10^7 – 10^8 PFU/ml) overnight. The preconditioned 5910 parental phenotypes are indicated as 5910 HIS', 5910 NHS' and 5910 Phage' according to the treatment. Whole genome sequencing and RNAseq data are available through the BioSample or BioProject (PRJNA1074513) accession numbers.

Strain	Precondition	Relevant characteristic	Capsule and LOS phenotype	Reference	BioSample accession number
5910 LB (parental strain)	LB	Variable	Variable	HUSLAB	SAMN39861904
5910 HIS'	25% HIS	Variable	Variable	HUSLAB	SAMN39861904
5910 NHS'	25%	Phage sensitive<br>Serum resistant	Encapsulated<br>Longer LOS	HUSLAB	SAMN39861904
5910 Phage'	Phage lysate	Phage resistant<br>Serum sensitive	Non-encapsulated<br>Shorter LOS	HUSLAB	SAMN39861904
5910-R_φ	LB	Phage resistant<br>Serum sensitive	Non-encapsulated<br>Shorter LOS	This study	SAMN39861905
5910-S_φ	LB	Phage sensitive<br>Serum resistant	Encapsulated<br>Longer LOS	This study	SAMN39861906

Table S2: Prediction of depolymerase activities in phage tail spike proteins. DNA sequences of the tail spike proteins of the phages fBenAci001, fBenAci002 and fBenAci003 were uploaded to the Phage Depolymerase Finder (PhageDPO) platform (Galaxy server of University of Minho, Version 0.1.0) for the prediction of potential depolymerases.

Phage	Accession number	Tail spike protein ID	Model DPO prediction [%]
fBenAci001	MW056501	QOV07748.1	99
fBenAci002	MW056502	QOV07800.1	100
fBenAci003	MW056503	QOV07848.1	99

Table S3: Sequence variance analysis of 5910-R$_φ$ and 5910-S$_φ$ clones. Location of mutations was identified in 5910-R$_φ$ and 5910-S$_φ$ clones through comparison with the parental 5910 strain by using the variant calling tool on PATRIC. Position: location of mutation relative to the parental 5910 strain. Reference: nucleotide(s) in the 5910 parental strain. Variance: nucleotide(s) in 5910-R$_φ$ and or 5910-S$_φ$ clones. Function: Coding sequence of the mutated gene. Type: Type of mutation on the of 5910-R$_φ$ and or 5910-S$_φ$ clones.

Clone with SNPs	Position	Reference	Variance	Function	Type
5910-R$_φ$, 5910-S$_φ$	1716213	G	A	Transposase	Synon
5910-R$_φ$, 5910-S$_φ$	2615135	TTC	TGGTC		Insertion
5910-R$_φ$, 5910-S$_φ$	2622815	CGGGCCG	CGGGGCCG	Mobile element protein	Insertion
5910-R$_φ$, 5910-S$_φ$	2624487	GCG	GGGCCG	Mobile element protein	Insertion
5910-R$_φ$, 5910-S$_φ$	2627418	GATAAAATC	TAAAAATC		Deletion
5910-S$_φ$	3412899	TGCAGCTTTAGCAG	TG	Polyribonucleotide nucleotidyltransferase (EC 2.7.7.8)	Deletion
5910-S$_φ$	3577262	T	C	hypothetical protein	Nonsyn
5910-S$_φ$	3577668	A	G	Transposase	Synon
5910-R$_φ$, 5910-S$_φ$	3590865	C	T	FIGfam050825	Nonsyn
5910-R$_φ$, 5910-S$_φ$	3613867	ATTTTTTCA	ATTTTCA		Deletion
5910-R$_φ$, 5910-S$_φ$	3614618	AGGCGA	AGGGCGA	TniB NTP-binding protein	Insertion
5910-R$_φ$, 5910-S$_φ$	3654572	A	G	SSU rRNA ## 16S rRNA, small subunit ribosomal RNA	
5910-R$_φ$, 5910-S$_φ$	811936	CACAGCAC	CC	Glycine dehydrogenase [decarboxylating] (glycine cleavage system P protein) (EC 1.4.4.2)	Deletion
5910-R$_φ$, 5910-S$_φ$	812143	GTGACCATTGACCATT	GTGACCATT	Glycine dehydrogenase [decarboxylating] (glycine cleavage system P protein) (EC 1.4.4.2)	Deletion
5910-R$_φ$, 5910-S$_φ$	812293	AGGCA	AGATGGCA	Glycine dehydrogenase [decarboxylating] (glycine cleavage system P protein) (EC 1.4.4.2)	Insertion
